# Multi-omics, multi-tissue analysis reveal role of extracellular matrix remodeling and sphingolipid homeostasis in edematous malnutrition (kwashiorkor)

**DOI:** 10.1186/s40364-026-01016-9

**Published:** 2026-10-28

**Authors:** Fabienne Nackers, Kirrily de Polnay, Ruben Almey, Simon Daled, Azahir Dan Djibo, Sarah De Saeger, Dieter Deforce, Maarten Dhaenens, Yannick Gansemans, Griet Glorieux, Ousmane Guindo, Benjamin Jenkins, Albert Koulman, Hans-Joerg Lang, Céline Langendorf, Debby Laukens, Christopher Mambula, Isabelle Mukagatare, Damon Parkington, Elh Ibrahim Tassiou, Wim Van Biesen, Tom Van De Wiele, Jill Van Massenhove, Filip Van Nieuwerburgh, Irène Adehossi, André Briend, Gerard Bryan Gonzales

**Affiliations:** 1 Epicentre, Paris, 75019 France; 2 Médecins Sans Frontières Operational Center of Brussels, Brussels, Belgium; 3 Laboratory of Pharmaceutical Biotechnology, Faculty of Pharmaceutical Sciences, Ghent University, Ghent, Belgium; 4 Epicentre, Maradi, Niger; 5 Centre of Excellence in Mycotoxicology and Public Health, Department of Bioanalysis, Faculty of Pharmaceutical Sciences, Ghent University, Ghent, Belgium; 6 Department of Internal Medicine and Paediatrics, Nephrology Section, Ghent University Hospital, Ghent, Belgium; 7 Wellcome Trust - MRC Institute of Metabolic Science Metabolic Research Laboratories, Addenbrooke’s Hospital, Hills Road, Cambridge, CB2 0QQ UK; 8 Medical Research Council Human Nutrition Research, Elsie Widdowson Laboratory, University of Cambridge, Fulbourn Road, Cambridge, CB1 9NL UK; 9 Heidelberg Institute of Global Health, University Heidelberg, Heidelberg, Germany; 10 NGO Alliance for International Medical Action/ALIMA, Dakar, Sénégal; 11 Department of Internal Medicine and Pediatrics, Ghent University, Ghent, Belgium; 12 VIB Center for Inflammation Research (IRC), Ghent University, Ghent, Belgium; 13 Ghent Gut Inflammation Group (GGIG), Ghent University, Ghent, Belgium; 14 Médecins Sans Frontières Operational Center of Paris, Paris, France; 15 Health District Medical Officer, Madarounfa, Niger; 16 Center for Microbial Ecology and Technology (CMET), Department of Biotechnology, Ghent University, Gent, Belgium; 17 Niamey National Hospital, Niamey, Niger; 18 Department of Nutrition, Exercise and Sports, Faculty of Science, University of Copenhagen, Frederiksberg, Denmark; 19 Tampere Center for Child, Adolescent and Maternal Health Research, Faculty of Medicine and Health Technology, Tampere University and Tampere University Hospital, Tampere, Finland; 20 Department of Public Health and Primary Care, Faculty of Medicine and Health Sciences, Ghent University, Ghent, Belgium

**Keywords:** Kwashiorkor, Edematous malnutrition, Extracellular matrix, Sphingolipid, Edema, Metabolomics, Proteomics, Lipidomics, Gut microbiome

## Abstract

**Background:**

Edematous malnutrition, aka kwashiorkor, is a phenotype of severe malnutrition whose pathophysiology remains poorly understood.

**Methods:**

In this case-control study, we employed plasma lipidomics, metabolomics, and proteomics, urine metabolomics and gut microbiome profiling to delineate molecular features specific to kwashiorkor in 60 children aged 6–59 months from Niger compared to marasmus (*n* = 60) and non-malnourished children (NAM) (*n* = 60) matched by age, sex, and clinical triage score. Features were defined as kwashiorkor-specific if they also correlated with edema severity and normalized following nutritional rehabilitation.

**Results:**

We identified a plasma proteomic factor featuring lumican, gelsolin, and tetranectin and a lipidomic factor enriched in sphingolipids. Both differed in kwashiorkor compared to marasmus and NAM, were associated with edema grade, and trended towards normalcy after nutritional treatment, which are consistent with ECM remodeling and sphingolipid dysregulation. In contrast, neither (plasma and urine) metabolomic profiles nor gut microbiome composition showed kwashiorkor-specific signatures, although our urine and gut microbiome analyses may be underpowered.

**Conclusion:**

These findings advance understanding of kwashiorkor pathophysiology beyond nutrient deficiency and highlight ECM and sphingolipid features associated with its distinct clinical and molecular characteristics compared to marasmus.

**Supplementary Information:**

The online version contains supplementary material available at https://doi.org/10.1186/s40364-026-01016-9.

## Background

Edematous malnutrition, also known as kwashiorkor, is a severe form of acute malnutrition characterized by bilateral edema, fatty liver, and skin changes. It is clinically and biochemically distinct from non-edematous malnutrition, also known as marasmus or severe wasting, evident as low weight-for-height (<−3 z-scores from the 2006 WHO standards), or a mid-upper arm circumference (MUAC) below 115 mm (children 6–59 months), visible muscle atrophy, and loose skin [1]. Both conditions may co-occur where a malnourished child presents with both severe wasting and edema – known as marasmic kwashiorkor. Kwashiorkor represents a critical public health issue in low-income countries although its actual prevalence is difficult to ascertain as it is often not captured in population-based surveys [2]. Despite its clinical recognition for over eight decades, the precise molecular mechanisms underlying the pathophysiology of kwashiorkor remain incompletely understood. This gap in knowledge impedes the development of targeted interventions and therapeutic strategies to effectively treat and prevent this devastating condition.

Previously, kwashiorkor had been attributed to protein deficiency [3]; however, emerging evidence suggests that its etiology is multifactorial, involving a complex interplay of nutritional, and environmental factors. Recent studies have highlighted the potential roles of oxidative stress [4], immune dysregulation [5], and alterations in the gut microbiome in the development of kwashiorkor [6]. Exogenous toxicants, including aflatoxins from staple foods like maize, groundnuts, sorghum, and millet often consumed in low and middle-incomes countries could also increase oxidative stress and explain the accumulation of liver fat in kwashiorkor. However, despite finding aflatoxins in biological samples of children with kwashiorkor [7–11], a causal link has not be demonstrated as the possibility of reverse causality has not been ruled out [12]. Additionally, the dysregulation of metabolic pathways, particularly those related to amino acid metabolism [13, 14], one-carbon metabolism [15], and the synthesis and breakdowns of extracellular matrix (ECM) proteins [16] and endothelial dysfunction [17], have been implicated in the disease process. Yet, the comprehensive molecular mechanisms remain to be fully elucidated.

To gain a deeper understanding of the molecular underpinnings of kwashiorkor, we employed a comprehensive multi-omics approach, integrating metabolomics, lipidomics, proteomics, and gut microbiome data across multiple biological samples throughout the different treatment stages to compare it to marasmus and non-malnourished children and explore its multifaceted pathophysiology.

## Methods

### Study design and participants

This matched case-control study was designed to analyze and compare biological specimens, including blood, urine, and feces, collected from children with kwashiorkor, marasmus, and non-acutely malnourished (NAM). Participants were matched by age categories, sex, and triage score as a proxy of clinical severity, with children classified into groups based on edema levels (mild, moderate, and severe), which served as an indicator of kwashiorkor severity (see Table 1).

**Table 1:** Recruitment groups and sample size

Kwashiorkor			Marasmus	Non-malnourished
+	20	Matching on: Age Sex Severity at baseline *(triage score)*	20	20
++	20		20	20
+++	20		20	20
Total	60		60	60

*(+) feet/ankles only, (++) feet/ankles and legs, (+++)on other part of the body*

Biological samples were collected at various time points [1]: upon recruitment for all study participants [2], at discharge from inpatient care, and [3] at exit from outpatient care (known in this context as an ambulatory therapeutic feeding center, ATFC). The collection frequency varied by participant group: NAM samples were collected once for children in outpatient care and at baseline and discharge from children in inpatient care, while kwashiorkor and marasmus groups underwent additional collections at the indicated discharge/exit points.

Collected samples (venous blood, stools and urine) were transported in cold chain to the Epicentre Research Laboratory in Maradi, Niger, where they were stored in preparation for shipment to Belgium, following a −80 °C cold chain protocol. Upon arrival, samples were distributed among collaborating laboratories for specific analyses, conducted according to the type of biological specimen and study objectives.

### Study setting

The study was conducted from the 26th of September 2018 to the 5th of November 2019 in the Madarounfa Health District of the Maradi region, located in a remote rural area of south-central Niger, along the Nigerian border. The local livelihood mostly relies on subsistence agriculture and animal farming. Acute childhood malnutrition is endemic in the area with seasonal peaks from May until October when the food security deteriorates before the harvest. In collaboration with the Ministry of Health of Niger, Médecins Sans Frontières (MSF) is supporting the pediatric ward, the level 1 intensive care unit and the inpatient therapeutic feeding center (ITFC) of the Madarounfa District hospital as well as five ambulatory therapeutic feeding centres (ATFC) in the district.

### Study population

This study recruited children with severe acute malnutrition aged 6 to 59 months eligible for new admission to either inpatient care in the ITFC of the Madarounfa District Hospital or enrolment in outpatient care in one of the ATFC supported by MSF in the region of Madarounfa. In addition, the study included non-acutely malnourished children from the paediatric ward of Madarounfa Health District Hospital or from the outpatient department of Madarounfa health centre. Children were classified according to their nutritional status and the severity of the oedema among children with kwashiorkor.

**Table 2:** Baseline characteristics and outcome of matched study participants

	Kwashiorkor (*n* = 60)	Marasmus (*n* = 60)	No acute malnutrition (NAM) (*n* = 60)
**Age (months) mean ± SD**	24.7 (7.8)	21.3 (9.0)	25.0 (12.1)
n (%) 6–11	2 (3.3)	11 (18.3)	6 (10.0)
12–23	27 (45.0)	20 (33.3)	24 (40.0)
24–35	24 (40.0)	26 (43.3)	17 (28.3)
36–47	5 (8.3)	2 (3.3)	8 (13.3)
48–59	2 (3.3)	1 (1.7)	5 (8.3)
**Sex (n female, %)**	36 (60.0)	36 (60.0)	36 (60.0)
Triage score* n (%)			
Green (non-urgent)	30 (50.0)	30 (50.0)	30 (50.0)
Yellow (priority)	29 (48.3)	29 (48.3)	29 (48.3)
Red (emergency)	1 (1.7)	1 (1.7)	1 (1.7)
**Initial recruitment site n (%)**			
**Children hospitalized**	30 (50)	30 (50)	30 (50)
**Children in ambulatory treatment facility n (%)**	30 (50)	30 (50)	30 (50)
**Clinical characteristics**			
**Level of edema at baseline** n (%)			
0	/	60 (100.0)	60 (100.0)
+	21 (35.0)	/	/
++	19 (31.7)	/	/
+++	20 (33.3)	/	/
**Middle upper arm circumference (mm)** Median [IQR]	122 [115, 130]	111 [107, 116.5]	139 [133, 145.5]
**Weight for height z-score** Median [IQR]	−2.14 [−2.84, −1.04]	−3.61 [−4.23, −3.20]	−1.26 [−1.60, −0.77]
**Weight for age z-score** Median [IQR]	−3.25 [−4.11, −1.94]	−4.16 [−4.91, −3.58]	−1.81 [−2.36, −1.03]
**Height for age z-score** Median [IQR]	−3.29 [−4.09, −2.06]	−2.88 [−4.59, −1.95]	−1.79 [−2.36, −0.71]
**PEWS at baseline n (%)**			
0–2 (green)	58 (96.7)	57 (95.0)	46 (76.7)
3–4 (yellow)	2 (3.3)	1 (1.7)	11 (18.3)
≥5 (orange/red)	0 (00.0)	2 (3.3)	3 (5.0)
**Temperature ≥ 38 (° C) n (%)**	3 (5.0)	8 (13.6)	18 (30.0)
**Severe clinical anaemia n (%)**	1 (1.7)	4 (6.7)	14 (23.3)
**Diarrhoea (≥3 stools) n (%)**	16 (26.7)	35 (58.3)	14 (23.3)
**O2 saturation ≥ 93% (ambient air) n (%)**	58 (98.3)	58 (98.3)	59 (98.3)
**Laboratory**			
**Malaria rapid test (n positive, %)**	32 (53.3)	29 (48.3)	41 (73.2)
**Glucose mg/dL - Median [IQR]**	79 [68,87]	88 [80,100]	91.5 [81,107]
**Haemoglobin g/dL - Median [IQR]**	9.2 [7;1,10.5]	9.9 [8.8,11.6]	9.2 [6.3,10.7]
**Haematocrit % - Median [IQR]**	27 [21,31]	30 [27,34]	27 [19,32]
**Creatinine mg/dL - Median [IQR]**	<0.2 [<0.2,0.2]	<0.2 [<0.2,<0.2]	<0.2 [<0.2,<0.2]
**ALT (U/L) - Median [IQR]**	35 [23,58]	18.5 [14,24]	20 [14,30]
**CRP (mg/L) - Median [IQR]**	13.1 [1.6,32.0]	12.4 [1.5,72.4]	72.9 [16.0,141]
**Total bilirubin (mg/dL) - Median [IQR]**	0.3 [0.2,0.4]	0.3 [0.2,0.4]	0.4 [0.2,1.1]
**Albumin (g/L) - Median [IQR]**	17.3 [13.2,21.5]	29.9 [21.4,34.1]	31.9 [26.2,36.6]
**Sodium mmol/L - Median [IQR]**	137 [134,140]	135 [131,137]	134 [132,137]
**Potassium mmol/L - Median [IQR]**	3.1 [2.7,3.8]	3.4 [2.7,4]	3.8 [3.3,4.2]
**Lactate mmol/L - Median [IQR]**	2.2 [1.3,3.1]	1.8 [1.3,2.6]	1.9 [1.4,3.1]
**Clinical progress and outcome**			
**Stay ≥ 1 days in intensive care n (%)**	2 (6.9)	3 (10.0)	4 (13.3)
**Final outcome (n %)**			
Cured	51 (85.0)	50 (83.3)	60 (100)
Deceased	2 (3.3)	0	0
Exit against medical advice	4 (6.7)	9 (15.0)	0
Not responding to nutrition treatment	3 (5.0)	1 (1.7)	/

* The study triage score was adapted from the Emergency triage Assessment and Treatment (ETAT) score applied in the MSF program in Niger at the time of the study. Details can be found in Supplementary table 2. Two nutritional criteria (visible severe wasting, oedema on both feet) were not included in the study triage score, wherein a malnourished child presenting in yellow (priority) case indicates that the child had another priority signs besides having visible severe wasting or oedema on both feet. PEWS: Pediatric Early Warning Scores; IQR: Interquartile range

### Nutritional status was defined as follows

- **Kwashiorkor** was defined by the presence of bilateral nutritional edema (mild to severe) - irrespective of either weight-for-height z-score (WHZ), mid-upper arm circumference (MUAC), presence of skin lesions, hair changes or other clinical features such as apathy, irritability, or fatty liver. The severity of edema was graded as mild (+) when it was present in feet/ankles only, as moderate (++) when it was present in feet/ankles and legs, and severe (+++) when it was visible on other part of the body (face, hands, arms, trunk etc) [18]. The presence of edema on feet/ankles was determined by applying moderate thumb pressure to the dorsum of both feet for at least three seconds. If bilateral pits remained after the thumbs have been removed, then the child would be considered as having nutritional edema. All children underwent a medical history assessment and a clinical examination (by two doctors for most hospitalized children). To avoid the inclusion of edema of non-nutritional origin, edematous children who could provide urine sample underwent a urine test strip and only children with negative results (i.e. negative for protein and blood) were included in the Kwashiorkor group. The presence of albumin in urine during renal disease/nephrotic syndrome contrasts with kwashiorkor where it is usually absent or present in very small amounts. Similarly, blood is found in nephritis but most often absent in kwashiorkor.
- **Marasmus,** or severe wasting, was defined as a WHZ less than −3 [19], and/or a MUAC less than 115 mm (in children 6-59 months) **and the absence of edema** (nutritional or not).
- **Non (acutely)-malnourished (NAM) status** was defined by the absence of acute malnutrition, that is: absence of nutritional edema, and a WHZ ≥ −2 and a MUAC ≥ 125 mm (in children 6-59 months).

### Inclusion criteria

Children were eligible for study participation when they fulfilled the following inclusion criteria:

- Aged 6 to 59 months at the time of inclusion.
- Diagnosed with severe acute malnutrition (kwashiorkor or marasmus) or non-acutely malnourished (see above definitions).
- Medical condition allowing the collection of 2 mL of blood in the 12 hours following admission and before any intravenous or intramuscular antibiotic treatment.
- Consent signed by the child’s parent (i.e. the mother or the father) or the child’s main caretaker if the child is an orphan.
- Newly admitted (i.e. not having been enrolled/admitted in the nutrition programme in the last 2 months nor in the study).
- Direct admission at the ITFC (i.e. not transferred to the ITFC while under treatment in an ATFC) or direct admission at the ATFC (i.e. not transferred from the ITFC).
- Child’s parent or main caretaker intending to remain with the child in the study area until the end of the study follow-up period.
- Absence of any exclusion criteria.

### Exclusion criteria

Children were excluded from participation in case of any of the following:

- Medical condition not allowing the collection of 2 mL of blood in the 12 hours following admission, based on the judgement of the clinician attending the child.
- Medical condition not allowing the collection of blood before any type of drug or fluid is administered via the intravenous (IV) catheter (when a catheter is placed upon admission), based on the judgement of the clinician attending the child.
- Intravenous or intramuscular antibiotics given in the 12 hours prior to the inclusion blood sampling. For artesunate injection, this was limited to 4 hours.
- Informed consent not provided.
- Previous enrolment/admission in the nutrition programme in the last 2 months (including relapse or return after default) or in the study.
- Current participation in another clinical research study including blood sampling and/or investigational products.
- Child’s parent or main caretaker not intending to remain with the child in the study area until the end of the study follow-up period.
- Current edema of non-nutritional origin (based on the judgement of the attending clinician or with positive urine test strip (for proteinuria ≥ 0.3 g/L (1+) and/or blood red cells or hemoglobin).
- Not “directly admitted” (e.g. children under treatment at the ATFC who are secondarily transferred to the ITFC due to clinical or nutritional deterioration; children transferred from the ITFC to the ATFC after nutritional stabilisation).
- Previous history of kwashiorkor (for patients admitted in the marasmus or non-malnourished groups).
- Known congenital abnormalities or a known chronic disease (including known tuberculosis, sickle cell disease, congenital heart disease …).
- Known HIV-positive status, clinical AIDS or having a known HIV-positive mother. [Considering the low HIV prevalence in Niger (estimated at 0.2% among adults 15-49 year-old in 2012 in the Maradi Region [20]), there was no systematic HIV screening].
- Admitted into inpatient care for trauma, burns, intoxication or surgery.

### Clinical biochemistry analyses

Urine sodium, potassium, chloride, and creatinine levels were measured using an Abbot Architect Clinical Chemistry Analyzer (Abbott, Illinois, USA). Urinary albumin was measured using the Beckman Paragon Electrophoresis system. Plasma triglycerides, cholesterol, HDL-cholesterol, total bilirubin, total protein, albumin, aspartate transferase (AST), alanine aminotransferase (ALT) and alkaline phosphatase (ALP) levels were determined using colorimetric methods and C-reactive protein (CRP) using a particle enhanced turbidimetric immunoassay (PETIA) technique all on a Siemens Dimension Xpand autoanalyzer (Siemens Healthcare, Camberley, UK) following manufacturer instructions. i-STAT point-of-care analyzer (Abbott Point of Care, Maidenhead, UK) used with the cartridge CHEM8+ (providing simultaneously glucose, kidney function [urea nitrogen, creatinine], hematology [hematocrit, hemoglobin], electrolytes [sodium, potassium, chloride, ionized calcium], anion gap) and the cartridge CG4+ (providing simultaneously lactate, blood Gases [PCO2, PO2, TCO2, sO2], pH, HCO3, base Excess).

### Plasma and urine untargeted metabolomics

Plasma samples were thawed on ice before metabolite extraction. Ice-cold methanol was used as the extraction solvent, added at a 2:1 ratio (1 mL solvent to 500 µL plasma). After thorough vortexing, the samples were stored at − 20 °C for one hour to allow protein precipitation. Following this, the samples were centrifuged at 13,000 × g for 10 minutes at 4 °C. The supernatant (750 µL) was transferred to a glass tube and dried under a gentle nitrogen stream at temperatures below 10 °C. The resulting dried residue was reconstituted in 10% acetonitrile containing 0.1% formic acid for subsequent liquid chromatography-mass spectrometry (LC-MS) analysis.

To create a quality control (QC) sample, 10 µL aliquots from each sample were pooled together. A dilution series of the QC sample was then prepared by mixing it with 0.1% formic acid at different ratios (1:1, 1:5, 1:10, and 1:50).

Chromatographic separation was performed on a Waters Acquity UPLC I-class FTN system (Waters, Manchester, UK), with dynamic mobile phase gradients and flow rates, as shown in our previously published paper [21] to optimize peak separation. The mobile phases consisted of 0.1% formic acid in water (A) and 0.1% formic acid in acetonitrile (B), with separation achieved using an Acquity UPLC HSS T3 column (1.8 µm, 100 Å, 1 mm × 100 mm). The injection volume was set at 5 µL, with the column maintained at 50 °C.

Liquid chromatography (LC) flow was directed into a Waters Synapt G2Si high-resolution mass spectrometer (MS) (Waters, Manchester, UK) using electrospray ionization (ESI) in both positive and negative modes. The capillary voltage was set at 2.75 kV for positive mode and 2.20 kV for negative mode. The source temperature was maintained at 150 °C, with a desolvation temperature of 500 °C. Gas flow rates were 20 L/h for the cone and 600 L/h for desolvation. The mass range covered 50–1000 Da, with a scan speed of 0.1 s in MS^E^ centroid resolution mode. MSE collision energy was ramped from 10 to 30 V for both modes. Mass calibration was achieved using sodium formate adducts, and online correction was applied with leucine-enkephalin (200 pg/µL), infused at 20 µL/min every 10 seconds.

A blank (100% acetonitrile) was injected 10 times at the beginning to condition the column followed by 10 injections of the QC sample. QC samples were re-injected after every 10 experimental samples. Experimental samples were analyzed in random order.

Data acquisition was monitored using MassLynx v1.7 (Waters, UK). Data processing: chromatogram peak picking, deconvolution and peak alignment were achieved using the proprietary Progenesis QI software (Nonlinear Dynamics, Newcastle, UK). Data preprocessing was performed in R. Features with missing values in ≥30% of samples were excluded. Remaining missing values were imputed with one-fifth of the minimum observed value for the corresponding feature. Feature intensities were natural-log transformed and autoscaled to zero mean and unit variance. Samples were subsequently ordered according to their analytical injection sequence. Signal-drift correction and quality-control filtering were performed using the *batchCorr* package [22] in R, based on pooled quality-control samples, a VVE covariance model, and model-based feature clustering. Features with a coefficient of variation (CV) ≥30% in quality-control samples were excluded.

### Plasma lipidomics

The lipid extraction was carried out using the Protein Precipitation Liquid Extraction Protocol (Jenkins, Ronis & Koulman, 2020). A 50 µL aliquot was taken from each plasma sample for the lipidomics analysis. To each aliquot, 650 µL of chloroform solution and 100 µL of LIPID Internal Standards (Lipid-IS) were added. The samples were vortexed, followed by the addition of 250 µL of methanol. After another round of vortexing, 400 µL of acetone was added. The samples were then vortexed again and centrifuged at ~20,000× g for 10 minutes to remove the insoluble material. The monolayer supernatant was carefully collected. The organic extracts (chloroform, methanol, and acetone) were dried using a Concentrator Plus system (Eppendorf, Stevenage, UK) for 60 minutes at 60 °C. The dried samples were reconstituted in 100 µL of a 2:1:1 mixture of propan-2-ol, acetonitrile, and water, and thoroughly mixed. The reconstituted samples were transferred into vials for analysis by LC-MS, and for each sample the volume of injection was 10 µL.

Chromatographic separation of lipids was performed using an HPLC system (Shimadzu UK Limited, Milton Keynes, UK). The stationary phase was a Waters Acquity UPLC® CSH C18 column (Waters, Hertfordshire, UK; 1.7 µm, I.D. 2.1 mm × 50 mm), maintained at 55 °C. The mobile phase consisted of two solutions: solution A (a 6:4 mixture of acetonitrile and water) and solution B (a 9:1 mixture of isopropanol and acetonitrile), both containing 10 mM ammonium formate. The separation was carried out at a flow rate of 0.6 mL/min and the gradient used followed the method described by (Jenkins, Ronis & Koulman, 2020). MS analysis was performed on a Thermo Scientific Exactive Orbitrap instrument with a heated electrospray ionization source (Thermo Fisher Scientific, Hemel Hempstead, UK). The ion source parameters were as follows: capillary temperature: 300 °C, source heater temperature: 420 °C, sheath gas flow: 40 (arbitrary), auxiliary gas flow: 15 (arbitrary), spare gas: 3 (arbitrary), source voltage: 4 kV. The mass spectrometer scan rate set at 4 Hz, giving a resolution of 25,000 (at 200 m/z) with a full-scan range of m/z 100 to 1800 with continuous switching between positive and negative mode.

The raw data were processed using MS-DIAL software (version 4.9), which included peak detection, alignment, and data annotation (Tsugawa et al., 2020). The following parameters were applied: Data collection: MS1 mass range: 120–1800 Da; retention time range: 0–10 min. Peak detection: minimum peak height: 10,000 amplitude units; mass window width: 0.1 Da; smoothing method: linear weighted moving average; smoothing level: 3 scans. Identification: retention time tolerance: 0.25 min; accurate mass tolerance: 0.006 Da; identification score threshold: 70%. Alignment: retention time tolerance: 0.25 min; MS1 tolerance: 0.005 Da.

Annotation of lipid molecular species was based on both accurate mass data acquired by mass spectrometry (MS) and specific retention times of each lipid from liquid chromatography (LC). The lipid database used was validated using Lipid Maps (http://www.lipidmaps.org/tools/ms). Lipid semi-quantification was performed using deuterated internal standards (Lipid-IS) for each lipid class, based on the known concentrations of the Lipid-IS added to each sample. This analytical process resulted in matrices containing the concentrations of annotated lipid molecular species, expressed in µM. Lipid features with missing values in more than 20% of samples were excluded. For each retained lipid, remaining missing values were imputed with one-fifth of the lowest observed value for that lipid. Lipid abundances were subsequently natural log transformed and auto-scaled by mean-centering and division by the standard deviation before statistical analysis.

### Plasma untargeted proteomics

Bottom-up proteomics sample preparation was done in fully randomized fashion using a urea-based in-sol digest. To 2 µL of neat plasma, 300 µL of denaturing buffer (8 M urea (Chem-Lab Analytical, Zedelgem, Belgium) in 1 M ammonium bicarbonate (Sigma-Aldrich, Saint Louis, USA)) was added, followed by three cycles of 3 min-ON/3 min-OFF sonication and vortexing. To reduce proteins, dithiothreitol (MP Biomedical, Irvine, USA) was added to a final concentration of 5 mM followed by incubation at 37 °C for 30 min. After cooling down to room temperature, alkylation was done using iodoacetamide (Sigma-Aldrich, Saint Louis, USA) added to a final concentration of 15 mM and incubation at room temperature for 30 min in the dark. Next, the tubes were diluted to 2 M urea with 1 M ammonium bicarbonate buffer and 3 µg of Trypsin/LysC (Promega, Madison, USA) was added to achieve an estimated 1:50 wt/wt ratio of enzyme to protein. This was incubated overnight at 37 °C after which resulting peptides were desalted using C18 reverse phase SPE cartridges (Strata-X, Phenomenex, Torrance, USA). The cartridges were conditioned by adding 1 mL of methanol (Biosolve, Valkenswaard, The Netherlands) and equilibrated with two additions of 1 mL ultrapure water. Samples were loaded slowly onto the cartridges and washed two times with 1 mL of 5% methanol in water before eluting in 1% formic acid (Chem-Lab Analytical, Zedelgem, Belgium) in methanol buffer and drying using a vacuum concentrator. Vacuum dried peptide samples were resuspended in 0.1% formic acid in LC-MS grade water (Biosolve, Valkenswaard, The Netherlands) and centrifuged at 16 000 x *g* for 10 min. The clear supernatant was transferred into vials and quantified using the Lunatic UV/Vis spectrophotometer (Unchained Labs, Pleasanton, USA). The resuspension volumes were then corrected to achieve a peptide concentration of 2 µg/µL in every sample vial. Finally, a quality control sample was prepared by pooling an equal volume from all samples.

Peptide samples were acquired in randomized order, alternating between sample and blank injections to prevent carry-over, and interspersed with a calibration standard (PepCal, Sciex, Massachusetts, USA) every 5 samples and quality control mixtures every 10 samples: 1 µL of sample was injected onto a Eksigent NanoLC 425 HPLC system (Sciex, Massachusetts, USA) equipped with a Triart C18 trapping column (5 mm × 0.5 mm, YMC) and a Luna Omega Polar C18 column (150 mm × 0.3 mm, particle size 3 µm, Phenomenex), operating in capillary flow mode (5 µL/min), coupled to a TripleTOF 6600+ mass spectrometer (Sciex, Massachusetts, USA) with the Optiflow TurboV ion source operating in positive mode. A 20-minute active gradient from 3 to 30% B was utilized, with mobile phases A and B consisting of 0.1% formic acid in water (Biosolve, Valkenswaard, The Netherlands) and 0.1% formic acid in acetonitrile (Biosolve, Valkenswaard, The Netherlands), respectively. Column temperature was maintained at 30 °C. A SWATH 99 variable window acquisition scheme with a total cycle time of 4 sec was used. MS1 spectra spanned a mass range of 400–1200 m/z in high sensitivity mode with 250 ms accumulation time and MS2 spectra were acquired in high sensitivity mode from 100 to 1500 m/z in 37.5 ms per scan. Ion source parameters were set to 4.5 kV for the ion spray voltage, curtain gas at 25 psi, nebulizer gas at 10 psi, heater gas at 20 psi, and 100 °C as source temperature. Instrument maintenance was required in the middle of the batch.

Peptide identification and quantification was done with DIA-NN 1.8 [23]. An iterative search strategy was employed to ensure maximum coverage of the proteome by first doing four separate searches starting from four different databases: (i) a predicted library from the UniProt human proteome [24] (reviewed only, retrieved on 06/01/2022) concatenated with the GPM cRAP database, (ii) a predicted library from the PeptideAtlas [25] human plasma build (2021–07), (iii) a deeply fractionated empirical DDA plasma library (ZenoTOF 7600, Sciex, Massachusetts, USA), (iv) a predicted library from an annotated secreted protein database, i.e., supplementary file 3 from Harney et al. [26]. For all searches, the following settings were used: enzyme Trypsin/P allowing for 2 missed cleavages, cysteine carbamidomethylation as fixed modification and up to 1 variable modification from methionine oxidation and protein N-term acetylation, peptide length of 6–35 with charge range 1–5, precursor m/z range 400–122, fragment ion m/z range 100–1500, MS1 accuracy of 12, MS2 accuracy of 25, scan window of 6, match-between-runs with smart profiling enabled and double-pass mode, heuristic protein inference based with proteotypicity definition based on gene names, and robust high precision quantification with normalization and MaxLFQ disabled. The four resulting spectral libraries were merged into one spectral library, which ended up containing 3529 protein isoforms, 2820 protein groups and 16,595 precursors in 13,930 elution groups. Finally, one search was performed on the entire dataset using the same settings as before but starting from the merged spectral library. The resulting precursor quantity matrix at a false discovery rate of 1% was subsequently used as input for data preprocessing.

First, failed injections (1 sample and 2 QC runs) were dropped from the dataset and (potential) contaminant peptides were filtered out (33 out of 9365 peptides). Peptides were then manually matched to all possible proteins of origin using the UniProt human proteome database as described previously. Peptide abundance values were log_2_ transformed and normalized using the RUV-III-C [27] algorithm to correct for instrument performance degradation. RUV-III-C used a grouping based on run order except for QC samples which were assigned to one group and peptides that differed most across the run order as determined from a two-sided unpaired t-test were used as negative controls. Finally, peptides not belonging to a smallest unique group were dropped and the normalized peptide abundances were summarized to the protein level using a robust summarization algorithm [28].

Proteins with missing values in ≥20% of samples were excluded. Remaining missing values were imputed using the minimum observed abundance of the corresponding protein. Batch effects were corrected using the parametric empirical Bayes ComBat method [29], with batch included as the adjustment variable and an intercept-only model. Proteins with ≥30% CV across quality-control samples were subsequently excluded, and quality-control samples were removed. Protein abundances were scaled across all timepoints by mean-centering and division by the standard deviation before statistical analysis.

### Fecal microbiome analysis

Total DNA was extracted from the pellet of 200 mg fecal matter by means of bead beating with a PowerLyzer (Qiagen, Venlo, the Netherlands) and phenol/chloroform extraction [30]. The beads were precipitated and removed after centrifugation at maximal speed for 5 min. The supernatant was transferred to new tubes, followed by extraction and purification with 500 µL phenol: chloroform: isoamilyc alcohol 25:24:1 (pH 7) and 700 μL chloroform. 450 µL supernatant was transferred to new tubes containing 500 µL ice-cold Isopropyl alcohol and added 45 µL 3 M sodium acetate, followed by cooling at −20 °C over 1 h. Isopropyl alcohol was discarded after centrifugation at maximum speed for 30 min and then air-dried DNA was re-suspended by adding 100 µL 1× TE buffer (10 mM Tris, 1 mM EDTA). The quality control of DNA extracts was performed by electrophoresis in a 1% (w/v) agarose gel (Life Technologies, Madrid, Spain).

For each sample, a sequencing library was constructed starting from 12.5 ng input gDNA according to the ‘16S Metagenomic Sequencing Library Preparation’ guide (Illumina). Briefly, 16S rRNA gene fragments were PCR-amplified from genomic DNA for 25 cycles using the 341F (5’-CCT ACG GGN GGC WGC AG −3’) and 785Rmod (5’-GAC TAC HVG GGT ATC TAA KCC-3’) [31] primer pair. The PCR amplicons were purified using Ampure XP beads (Beckman Coulter). Sequencing adapters were added using the Nextera XT Index Kit (Illumina) with 8 PCR cycles and the resulting sequencing libraries were purified on Ampure XP beads. Quality was checked on a Bioanalyzer using a DNA 1000 chip (Agilent) and quantification was done using qPCR according to the ‘Sequencing Library qPCR Quantification Guide’ from Illumina. Finally, libraries were pooled equimolarly, spiked with 20% PhiX and sequenced as paired-end 150 on a MiSeq device (Illumina).

Data preparation and metagenomics analyses were done using QIIME2 (v2020.2) [32]. For each sample, read pairs were quality-trimmed, denoised and reconstructed into 16S amplicons using the DADA2 algorithm. Operational taxonomic units (OTU) and their abundance were collected. Taxonomic assignment was done using the Greengenes 16S reference collection [33] (release 13.8). These were then used to calculate alpha diversity metrics (Shannon, Faith Phylogenetic Distance, Pielou Evenness, Observed OTUs) and beta diversity metrics (Weighted and Unweighted UniFrac, Bray-Curtis, Jaccard). Alpha diversity metrics were used to detect differences between sample groups (Kwashiorkor, Marasmus, NAM) by means of Kruskal-Wallis tests. Group comparisons using beta diversity metrics were done with the PERMANOVA module in QIIME2. We used the ANCOM module to identify differentially abundant features across sample groups at the taxonomic level of family, genus and strain. We used OTU abundancies with the full pipeline of the PICRUSt2 module to perform a functional composition analysis at the taxonomic family, genus and strain levels, based on KEGG orthologs, Enzyme Classification numbers (EC) and MetaCyc pathways.

### Statistical analysis

In this study, we imposed strict criteria for the determination of kwashiorkor-associated biomarkers. Inferences on individual lipids, metabolites and proteins were obtained. Moreover, we also obtained inferences at the feature cluster level by consolidating correlated metabolites, lipid and proteins into uncorrelated latent variables (LV) using factor analysis (FA). Each omics domain was first analyzed separately. The use of factor analysis affords us to reduce the big omics datasets into explainable LVs, which can be used for further downstream analyses. Each LV represents a statistically derived module of correlated molecular features. LVs should not be interpreted as biological pathways directly; rather, they summarize coordinated variation among groups of proteins, lipids, or metabolites that may reflect shared biological regulation or related biological functions. Biological interpretation of each LV was performed post hoc by examining the identity and known functions of the features included therein, which were linked to known pathways (using DAVID [34] for proteomics) or known classes (especially lipidomics). The advantage of using LVs is that it provides a singular value that potentially refer to an overall behavior of the particular LV. This allowed us to make inferences on feature cluster level, instead of the traditional single feature analysis, typical of omics studies.

Features fulfilling the following criteria were considered candidate biomarkers of kwashiorkor. The study design does not permit inference regarding causality or whether these biomarkers represent upstream drivers, downstream consequences, or parallel manifestations of disease processes:

Criterion 1. The feature or LV must be different in kwashiorkor compared to marasmus and NAM at recruitment. Marasmus and NAM are grouped together in subsequent analyses. Analyses are matched for age, sex and clinical triage score. This approach was chosen to identify molecular features specific to kwashiorkor rather than to acute malnutrition in general. Consequently, candidate biomarkers were required to distinguish kwashiorkor from both severe undernutrition without edema (marasmus) and children without acute malnutrition (NAM).

Criterion 2. The feature or LV must be associated with the severity of edema (+, ++, +++) at recruitment.

Criterion 3. The feature or LV must change towards normalcy as the child with kwashiorkor recovers to a non-malnourished state following nutritional therapy.

These strict criteria ensure that our findings are robust despite the small sample size of this study (*n* = 180). However, analyses of the urine metabolome and gut microbiome only followed criteria 1 and 2 since these analyses were only performed on samples obtained at baseline.

### Univariable analyses

Scales and log-transformed levels of individual metabolite, lipid and protein features were compared between kwashiorkor and combined marasmus and NAM using conditional logistic regression (*clogit*) analysis to account for the age, sex and clinical severity matching. Significance was accepted at *p* < 0.05 after Benjamini Hochberg False Discovery Rate (FDR) correction [35]. Significant features were then forwarded to succeeding analysis assessing their individual levels association with severity of edema using ordinal regression using the *polr* function of the MASS package in R. Features showing a relationship with edema severity where assessed for their change over time using linear mixed model, where the features were tested for association with timepoint, age and sex, with subject as random effect. This was done using the *lmerTest* [36] package in R.

### Factor analysis

Latent variables (LV) were calculated separately for the metabolome, lipidome and proteome for the samples collected at baseline. Factor analysis was performed using the matrix-free likelihood method of extraction followed by the “varimax” rotation using the *fad* [37] package in R using the preprocessed omics data explained above (log transformed and unit scaled). The *fad* package was selected because it accommodates high-dimensional datasets in which the number of features far exceeds the number of participants. The Bayesian information criterion (BIC) was used to determine the optimal number of latent variables to extract. To facilitate interpretation, features with an absolute factor loading ≥ 0.40 were considered representative of an LV. The LVs were derived exclusively from the baseline omics datasets. The resulting factor loading matrices were then kept fixed and used to calculate factor scores for samples collected at hospital discharge and at the end of nutritional rehabilitation/recovery. Consequently, the factor scores at discharge and recovery represent projections of these samples onto the same latent biological dimensions identified at baseline. This approach ensures that a given LV corresponds to the same combination of molecular features across all timepoints, thereby allowing meaningful longitudinal comparison of factor scores.

Factor scores were then calculated from these variables using the *psych* package in R [38], using the Bartlett method [39], which enabled the fixed baseline loading matrices to be applied to the other timepoints. Thus, scores at discharge and recovery represent projections of these samples onto the same latent dimensions identified at baseline. The use of *psych* for factor scoring did not alter the factor solution estimated using *fad*. Factor scores were calculated for the different timepoints: baseline, hospital discharge and full nutritional and clinical recovery. These factor scores were then used for subsequent analysis to represent the entire LV, either for metabolome (mLV), proteome (pLV) or lipidome (lLV).

The internal stability of the baseline factor solution was assessed using 1,000 participant-level bootstrap samples, generated by resampling participants with replacement within clinical groups to preserve the original group sizes. Factor analysis was repeated in each bootstrap sample using the same number of factors, matrix-free likelihood extraction method, and varimax rotation as in the primary analysis. Bootstrap factors were matched to the original solution by optimal one-to-one assignment and sign alignment. Similarity was quantified using Tucker’s congruence coefficient [40].

mLV, lLV and pLVs were compared between kwashiorkor and combined marasmus and NAM using conditional logistic regression (*clogit*) analysis to account for the age, sex and clinical severity matching. Significance was accepted at *p* < 0.05 after Benjamini Hochberg False Discovery Rate (FDR) correction [35]. Significant LVs were then forwarded to succeeding analysis assessing their individual levels association with severity of edema using ordinal regression using the *polr* function of the MASS package in R. LVs showing a relationship with edema severity were assessed for their change over time using linear mixed model, where the factors were tested for association with timepoint, age and sex, with subject as random effect. This analysis was only done within the kwashiorkor group, and was performed using the *lmerTest* [36] package in R.

As sensitivity analyses, the analyses were reperformed after removing children with malaria and again after only including children with negative blood or protein in the urine measured using a urine test strip.

### Partial correlation network integration

Integration of the different omics domains were performed at the LV level using partial correlation network analysis using the *qgraph* [41] package in R. mLV, lLV and pLV were combined with clinical biochemistry results to show inter-LV association with known clinical markers.

## Results

### Participant characteristics

A total of 180 children aged 6–59 months were enrolled in the study, with kwashiorkor (*n* = 60), marasmus (*n* = 60) and without acute malnutrition (*n* = 60) matched on sex, age and clinical triage score. Majority (60%) of the children were female and age categories were evenly distributed at baseline (50% 6–23 months, 50% 24–59 months old). An even distribution was accomplished between the edema severity levels on baseline, except for one child who was recruited as presenting moderate edema (++) but was secondarily reconsidered as having low severity edema (+). Of the 60 kwashiorkor participants, 12 had at least one type of cutaneous lesion, and these children with skin lesions all had moderate to severe edema (++/+++), except for one child with the lowest edema severity score (+).

Height-for-age z-scores indicated a substantial burden of stunting. Most children were not critically ill (Table 3 and Supplementary table 1), with only one child per group classified as an emergency case based on study triage score at admission. NAM children were more likely to test positive for malaria using rapid diagnostic tests compared to children with kwashiorkor or marasmus and they also presented a higher Pediatric Early Warning Scores (PEWS) at baseline. Marasmic children suffered more commonly from diarrhea. Laboratory findings upon admission for the three groups are compared in the Table 1 and additional clinical characteristics and socioeconomic indicators of the participants can be found in Supplementary table 1. Two kwashiorkor participants died.

**Table 3:** Baseline characteristics and outcome of matched study participants

	Kwashiorkor (*n* = 60)	Marasmus (*n* = 60)	No acute malnutrition (NAM) (*n* = 60)
Age (months) mean ± SD	24.7 (7.8)	21.3 (9.0)	25.0 (12.1)
n (%) 6–11	2 (3.3)	11 (18.3)	6 (10.0)
12–23	27 (45.0)	20 (33.3)	24 (40.0)
24–35	24 (40.0)	26 (43.3)	17 (28.3)
36–47	5 (8.3)	2 (3.3)	8 (13.3)
48–59	2 (3.3)	1 (1.7)	5 (8.3)
Sex (n female, %)	36 (60.0)	36 (60.0)	36 (60.0)
Triage score* n (%)			
Green (non-urgent)	30 (50.0)	30 (50.0)	30 (50.0)
Yellow (priority)	29 (48.3)	29 (48.3)	29 (48.3)
Red (emergency)	1 (1.7)	1 (1.7)	1 (1.7)
Initial recruitment site n (%)			
Children hospitalized	30 (50)	30 (50)	30 (50)
Children in ambulatory treatment facility n (%)	30 (50)	30 (50)	30 (50)
Clinical characteristics			
Level of edema at baseline n (%)			
0	/	60 (100.0)	60 (100.0)
+	21 (35.0)	/	/
++	19 (31.7)	/	/
+++	20 (33.3)	/	/
Middle upper arm circumference (mm) Median [IQR]	122 [115, 130]	111 [107, 116.5]	139 [133, 145.5]
Weight for height z-score Median [IQR]	−2.14 [−2.84, −1.04]	−3.61 [−4.23, −3.20]	−1.26 [−1.60, −0.77]
Weight for age z-score Median [IQR]	−3.25 [−4.11, −1.94]	−4.16 [−4.91, −3.58]	−1.81 [−2.36, −1.03]
Height for age z-score Median [IQR]	−3.29 [−4.09, −2.06]	−2.88 [−4.59, −1.95]	−1.79 [−2.36, −0.71]
PEWS at baseline n (%)			
0–2 (green)	58 (96.7)	57 (95.0)	46 (76.7)
3–4 (yellow)	2 (3.3)	1 (1.7)	11 (18.3)
≥5 (orange/red)	0 (00.0)	2 (3.3)	3 (5.0)
Temperature ≥ 38 (° C) n (%)	3 (5.0)	8 (13.6)	18 (30.0)
Severe clinical anaemia n (%)	1 (1.7)	4 (6.7)	14 (23.3)
Diarrhoea (≥3 stools) n (%)	16 (26.7)	35 (58.3)	14 (23.3)
O2 saturation ≥ 93% (ambient air) n (%)	58 (98.3)	58 (98.3)	59 (98.3)
Laboratory			
Malaria rapid test (n positive, %)	32 (53.3)	29 (48.3)	41 (73.2)
Glucose mg/dL - Median [IQR]	79 [68,87]	88 [80,100]	91.5 [81,107]
Haemoglobin g/dL - Median [IQR]	9.2 [7;1,10.5]	9.9 [8.8,11.6]	9.2 [6.3,10.7]
Haematocrit % - Median [IQR]	27 [21,31]	30 [27,34]	27 [19,32]
Creatinine mg/dL - Median [IQR]	<0.2 [<0.2,0.2]	<0.2 [<0.2,<0.2]	<0.2 [<0.2,<0.2]
ALT (U/L) - Median [IQR]	35 [23,58]	18.5 [14,24]	20 [14,30]
CRP (mg/L) - Median [IQR]	13.1 [1.6,32.0]	12.4 [1.5,72.4]	72.9 [16.0,141]
Total bilirubin (mg/dL) - Median [IQR]	0.3 [0.2,0.4]	0.3 [0.2,0.4]	0.4 [0.2,1.1]
Albumin (g/L) - Median [IQR]	17.3 [13.2,21.5]	29.9 [21.4,34.1]	31.9 [26.2,36.6]
Sodium mmol/L - Median [IQR]	137 [134,140]	135 [131,137]	134 [132,137]
Potassium mmol/L - Median [IQR]	3.1 [2.7,3.8]	3.4 [2.7,4]	3.8 [3.3,4.2]
Lactate mmol/L - Median [IQR]	2.2 [1.3,3.1]	1.8 [1.3,2.6]	1.9 [1.4,3.1]
Clinical progress and outcome			
Stay ≥ 1 days in intensive care n (%)	2 (6.9)	3 (10.0)	4 (13.3)
Final outcome (n %)			
Cured	51 (85.0)	50 (83.3)	60 (100)
Deceased	2 (3.3)	0	0
Exit against medical advice	4 (6.7)	9 (15.0)	0
Not responding to nutrition treatment	3 (5.0)	1 (1.7)	/

* The study triage score was adapted from the Emergency triage Assessment and Treatment (ETAT) score applied in the MSF program in Niger at the time of the study. Details can be found in Supplementary table 2. Two nutritional criteria (visible severe wasting, oedema on both feet) were not included in the study triage score, wherein a malnourished child presenting in yellow (priority) case indicates that the child had another priority signs besides having visible severe wasting or oedema on both feet. PEWS: Pediatric Early Warning Scores; IQR: Interquartile range

### Integrated plasma lipidome, metabolome, proteome and clinical biochemistry of children with kwashiorkor, marasmus and non-acutely malnourished

#### Plasma proteomics

**Fig. 1:**
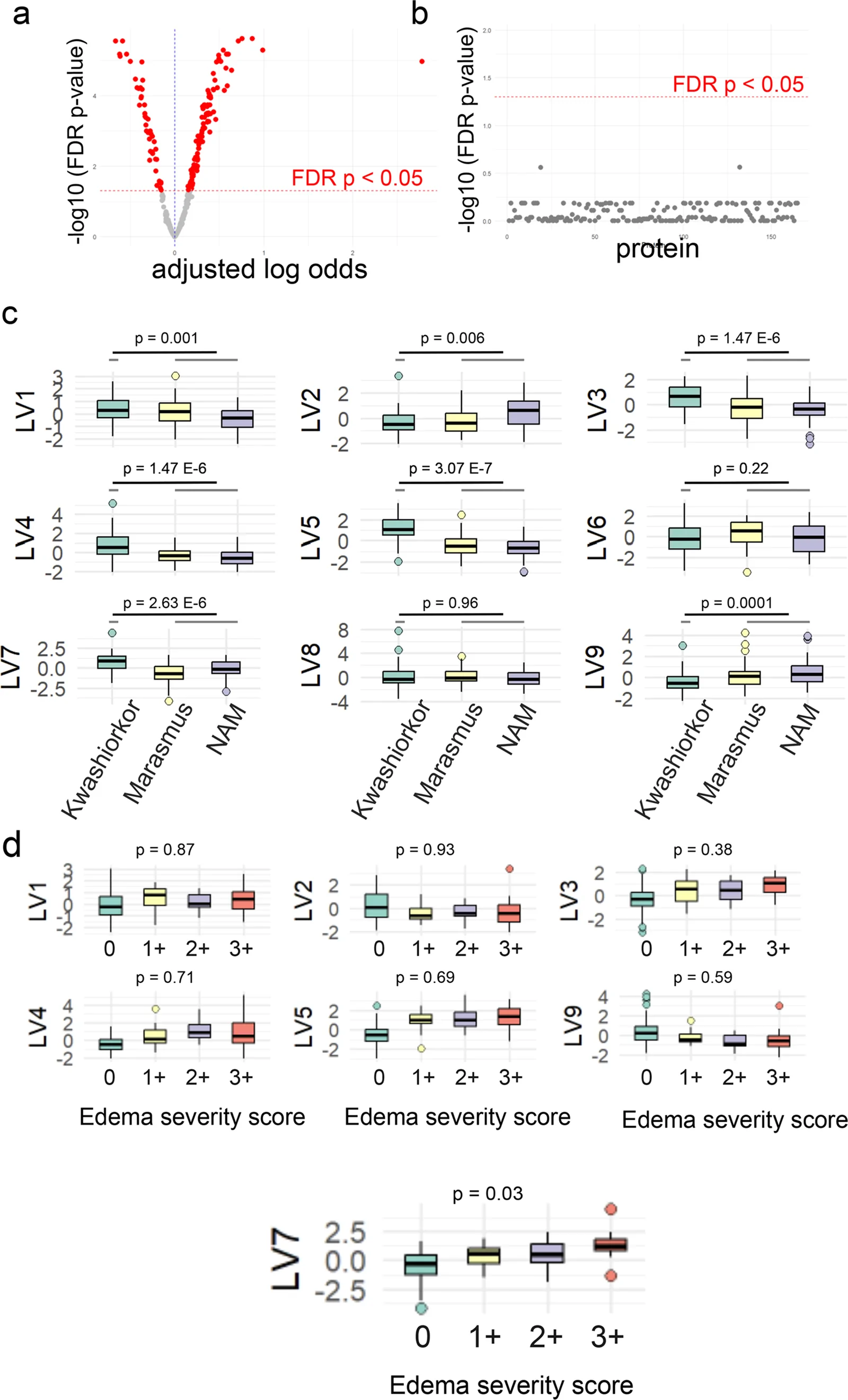

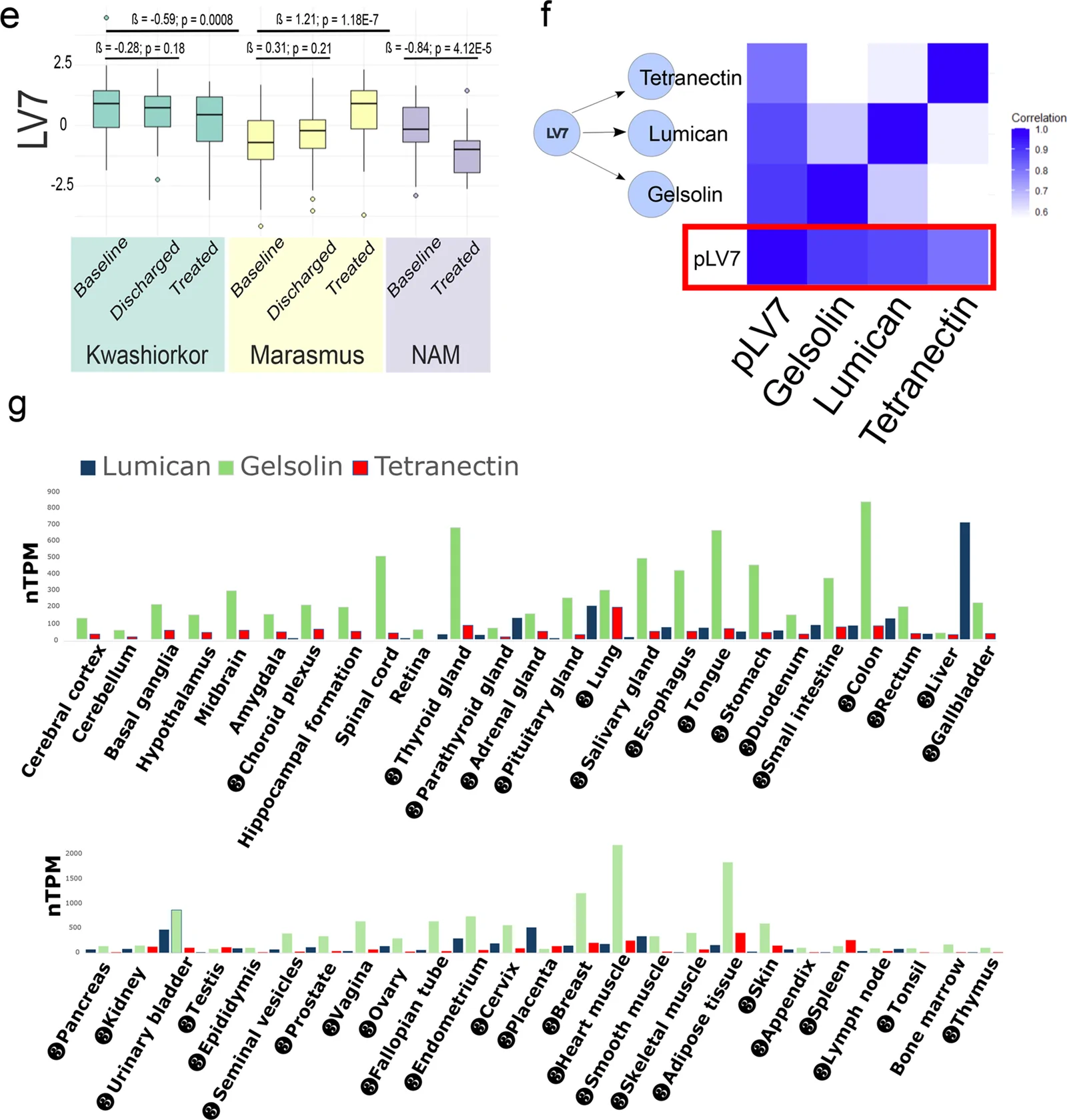
Proteomics analysis of plasma samples from children with kwashiorkor, marasmus and non-acutely malnourished (NAM) at baseline, discharge from hospital and treated (either end of nutritional rehabilitation for malnourished children or end of treatment for hospitalized NAM). (**a**) Univariable analysis of proteins at baseline (*n* = 180). (**b**) Proteins showing difference at FDR *p* < 0.05 were tested for association with edema severity among children with kwashiorkor (*n* = 60). (**c**) proteome-related latent variables (pLV) associated with kwashiorkor, showing 7 out of 9 pLvs with difference at FDR *p* < 0.05 in kwashiorkor compared to marasmus and NAM combined. (**d**) The 7 pLvs were tested for association with edema severity among children with kwashiorkor, where only pLV7 remained of interest (OR = odds ratio). (**e**) Trend of pLV7 at baseline (*n* = 180), discharge from hospital (*n* = 60) and at the end of ambulatory nutritional rehabilitation for kwashiorkor and marasmus (*n* = 90), and end of treatment for NAM (*n* = 30) – collectively called *treated*. pLV7 is significantly reduced at treatment compared to baseline among children with kwashiorkor. (**f**) Annotation of proteins involved in pLV7 showing the correlation of the individual proteins with pLV7. Data for the individual proteins and LVs across the different groups and time points can be visualized in this link: https://mudiboevans.Shinyapps.io/MSFKwash/. (**g**) Tissue localization of the pLV7-component proteins using the human proteome atlas (https://www.Proteinatlas.org/) [42]. Organs labeled with ❸ are those where all three proteins (lumican, gelsolin and tetranectin) are expressed. nTPM = normalized transcripts per million [42]

Untargeted proteomics analysis revealed 121 proteins were upregulated and 44 were downregulated (false discovery rate [FDR] *p*-value < 0.05) out of 308 protein features in kwashiorkor compared to both marasmus and NAM combined (Fig. 1a). However, when tested for association with edema severity in kwashiorkor, none of these proteins were found to have passed criterion 2 (Fig. 1b).

FA was then employed to determine clusters of proteins that share a latent relationship, potentially belonging to the same pathway. FA reduced the proteomics data into 9 proteome-LVs (pLV). Component proteins in each LV are shown in Supplementary file 1. Figure 1c shows the differences in the pLVs between kwashiorkor and non-kwashiorkor (marasmus and NAM). Out of 9 pLVs, 7 were found to be different in kwashiorkor (pLV 1, 3, 4, 5 and 7 were higher in kwashiorkor, pLV2 and 9 were lower in kwashiorkor than non-kwashiorkor cases), and hence passed criterion 1. Regression analyses of these pLVs with kwashiorkor edema severity revealed that only pLV7 was significantly associated with increasing severity of edema in kwashiorkor at baseline (Fig. 1d). Moreover, this pLV was also higher than in non-kwashiorkor children at baseline, but this level significantly reduced during and after nutritional therapy (Fig. 1e), passing all 3 criteria for a candidate pathway associated with kwashiorkor.

Uncovering the component proteins of pLV7 revealed that the extracellular matrix proteins lumican, gelsolin and tetranectin were involved in kwashiorkor pathophysiology. The internal stability of the factor solution was assessed by bootstrapping 1,000 participant-level samples and aligned each resampled solution to the original loading matrix. The complete pLV7 loading pattern showed modest stability, with a mean Tucker congruence coefficient of 0.67 [median: 0.71; 95% bootstrap interval: 0.25–0.90]. This was largely attributed to several proteins showing poor retention in the LV. However, when stability was evaluated specifically for this three-protein core, the mean Tucker congruence coefficient was 0.95 [median: 0.99; 95% bootstrap interval: 0.48–0.99]. Lumican, gelsolin, and tetranectin retained absolute loadings ≥ 0.40 in 80, 72, and 73% of bootstrap samples, respectively, with sign consistency of 99, 98, and 95%. These findings indicate that the three-protein ECM-related core was consistently recovered in pLV7.

The high correlation between these individual proteins and pLV7 demonstrates that pLV7 was able to capture the behavior of these three proteins as a cluster (Fig. 1f). Using the Human Proteome Atlas [42], these three proteins appear to be found in many tissue types all over the body, and can be found co-expressed in 41 out of 50 human organs (Fig. 1g). However, the organs where they are most highly expressed varied – lumican in gall blader, gelsolin in heart muscle, tetranectin in adipose tissue.

Because the NAM children were more likely to have a positive malaria rapid test on admission (Supplementary table 3), a first sensitivity analysis was conducted wherein children testing positive for malaria rapid diagnostic test were removed. Also, because not all children with kwashiorkor could produce urine on admission, and hence some were included without a urine strip result (Supplementary table 3), a second sensitivity analysis was conducted including only kwashiorkor children who tested negative for urine blood and protein. Both revealed that the association of pLV7 and kwashiorkor is robust, albeit with lower power due to the smaller sample size (especially for criterion 2) (Supplementary figure 1).

#### Plasma lipidomics

**Fig. 2:**
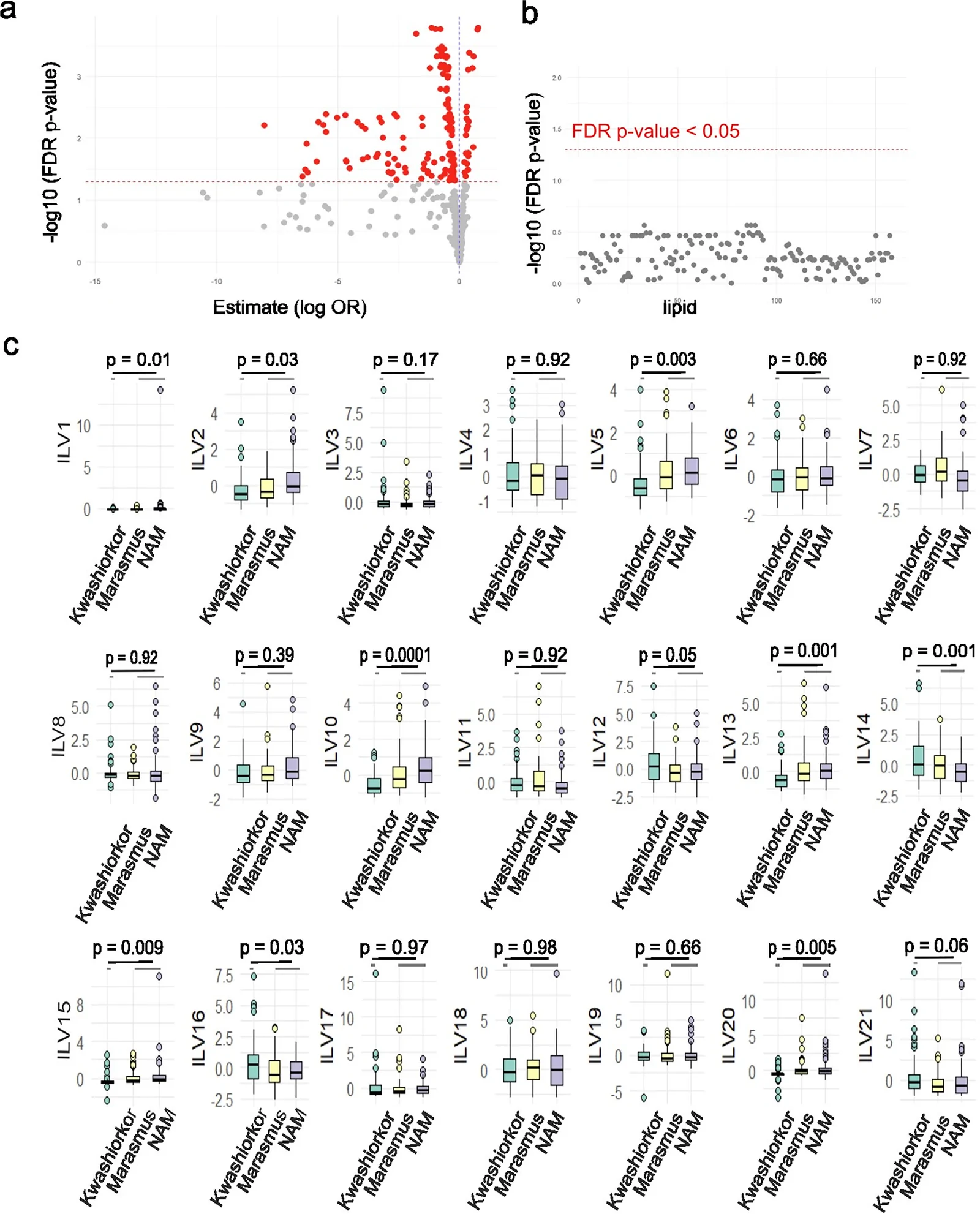

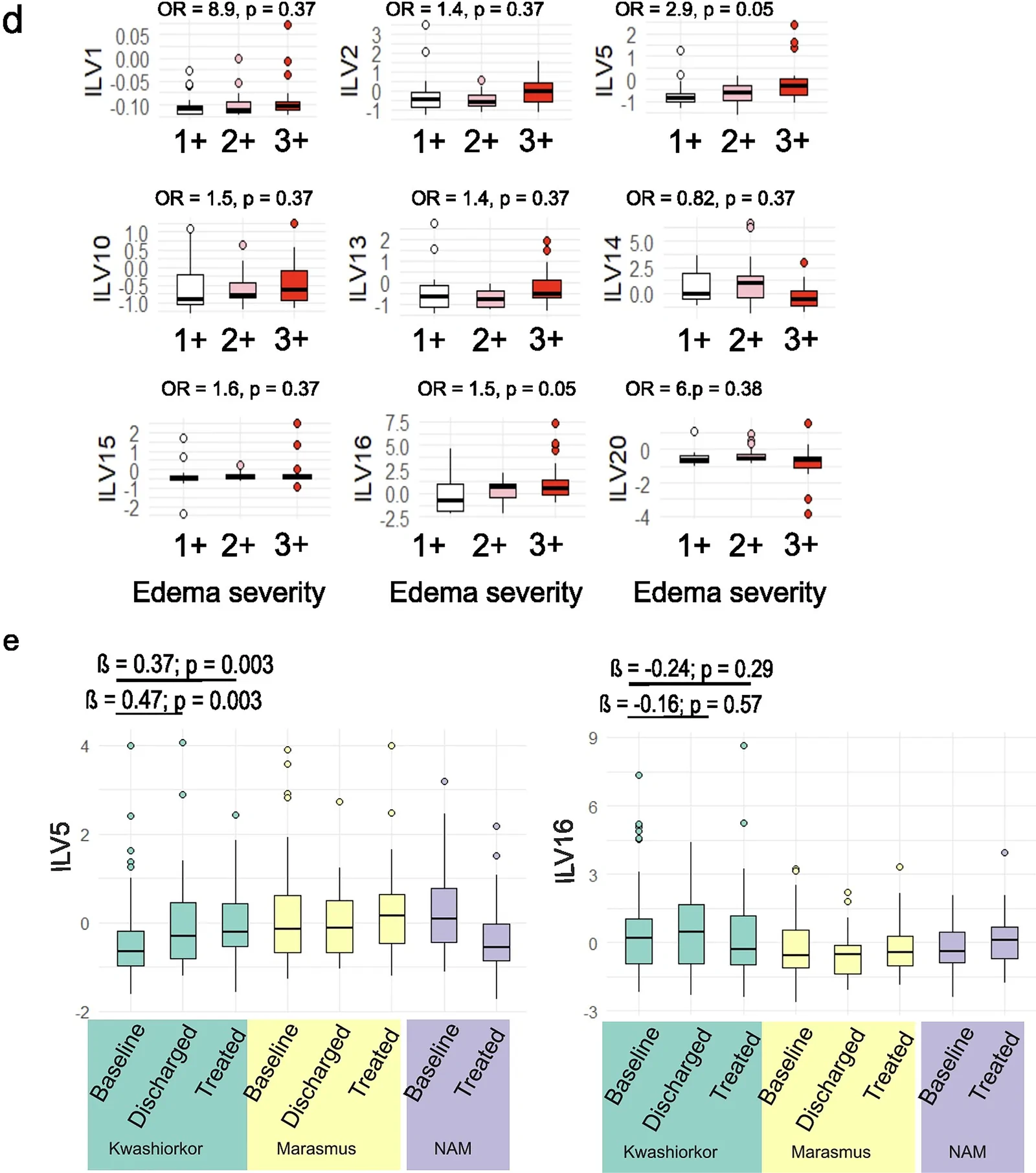
Lipidomics analysis of plasma samples from children with kwashiorkor, marasmus and non-acutely malnourished (NAM) at baseline, discharge from hospital and treated (either end of nutritional rehabilitation for malnourished children or end of treatment for NAM). (**a**) Univariable analysis of lipids at baseline (*n* = 180). (**b**) Lipids showing difference at FDR *p* < 0.05 were tested for association with edema severity among children with kwashiorkor (*n* = 60). (**c**) Lipidome-related latent variables (lLV) associated with kwashiorkor, showing 9 out of 21 lLvs with difference at FDR *p* < 0.05 in kwashiorkor compared to marasmus and NAM combined. (**d**) The 9 lLvs were tested for association with edema severity among children with kwashiorkor, where only lLV5 and lLV16 remained of interest (OR = odds ratio). (**e**) Trend of level of lLV5 and 16 at baseline (*n* = 180), discharge from hospital (*n* = 64) and at the end of ambulatory nutritional rehabilitation for kwashiorkor and marasmus (*n* = 90), and end of treatment for NAM – collectively called *treated* (*n* = 30). lLV5 is significantly increased at treatment compared to baseline among children with kwashiorkor. Group and time differences of the individual lipids in lLV5 is shown in Supplementary figure 3. Data for all the individual lipids and lLvs across the different groups and time points can be also visualized in this link: https://mudiboevans.Shinyapps.io/MSFKwash/

Untargeted lipidomics analysis of plasma revealed 22 lipids were upregulated and 136 were downregulated (FDR *p*-value < 0.05) out of 506 lipid features in kwashiorkor compared to both marasmus and NAM combined (Fig. 2a). However, when assessed for their association with severity of edema in kwashiorkor, none of these lipids were found to have passed criterion 2 (Fig. 2b). Hence, no individual lipid species was found to be strongly associated with kwashiorkor in this study.

FA reduced the lipidomics data into 21 lipid-LVs (lLV). Component lipids in each LV is shown in Supplementary file 1. Bootstrapping 1,000 participant-level samples revealed that lLV5 loading pattern showed strong stability, with a mean Tucker congruence coefficient of 0.91 [median: 0.94; 95% bootstrap interval: 0.57–0.98], with mean retention of 78% and mean sign consistency of 99%. Fig. 2cshows the differences in the lLVs between kwashiorkor and non-kwashiorkor (marasmus and NAM). Out of 21 lLVs, 9 were found to be different in kwashiorkor (lLV 14 and 16 were higher in kwashiorkor, lLV 1, 2, 5, 10, 13, 15 and 20 were lower in kwashiorkor than non-kwashiorkor cases), and hence passed criterion 1. Regression analyses of these lLVs with kwashiorkor edema severity revealed that lLV5 and lLV16 were significantly associated with increasing severity of edema in kwashiorkor at baseline (Fig. 2d). However, only lLV5 was significantly changed throughout the course of the treatment (Fig. 2e). At baseline, this lLV was lower in kwashiorkor than non-kwashiorkor cases, and its level increased in plasma after treatment making this lLV a strong candidate biomarker signature associated with kwashiorkor.

The component lipids of lLV5 are dominated by 17 mono- to di-unsaturated sphingomyelins (SM 35:1, 37:2, 38:1, 38:2, 39:1, 40:0, 40:1, 40:2, 41:0, 41:1, 42:0, 42:1, 42:2, 43:1, 44:0, 44:1, and 44:2), along with 5 polyunsaturated cholesterol esters (16:2, 18:3, 20:4, 20:5, 22:6), ganglioside GD3 (42:1), four saturated hexosylceramides (HexCer 39:0;O3; HexCer 40:0;O3: HexCer 41:0;O3 and HexCer 42:0;O3), Ether-linked lysophosphatidylcholine (LPC O-24:0) and phosphatidylcholines (PC 20:0, PC 22:0, PC 22:3, PC 37:1). Group and time comparison of individual lipid components of lLV5 are presented in Supplementary figure 3. The correlation between lLV5 and all its individual lipid components are shown in a heatmap in Supplementary figure 4. We show that all component lipids of lLV5 are strongly positive correlated at admission, showing good representation of lLV5 of its components.

Because the NAM children were more likely to have a positive malaria rapid test on admission (Supplementary table 3), a first sensitivity analysis was conducted wherein children testing positive for malaria rapid diagnostic test were removed. Also, because not all children with kwashiorkor could produce urine on admission, and hence some were included without a urine strip result (Supplementary table 3), a second sensitivity analysis was conducted including only kwashiorkor children with negative blood and protein urine test. Both revealed that the association of lLV5 and kwashiorkor is robust, albeit with lower power due to the smaller sample size (especially for criterion 2) (Supplementary figure 2).

#### Plasma metabolomics

**Fig. 3:**
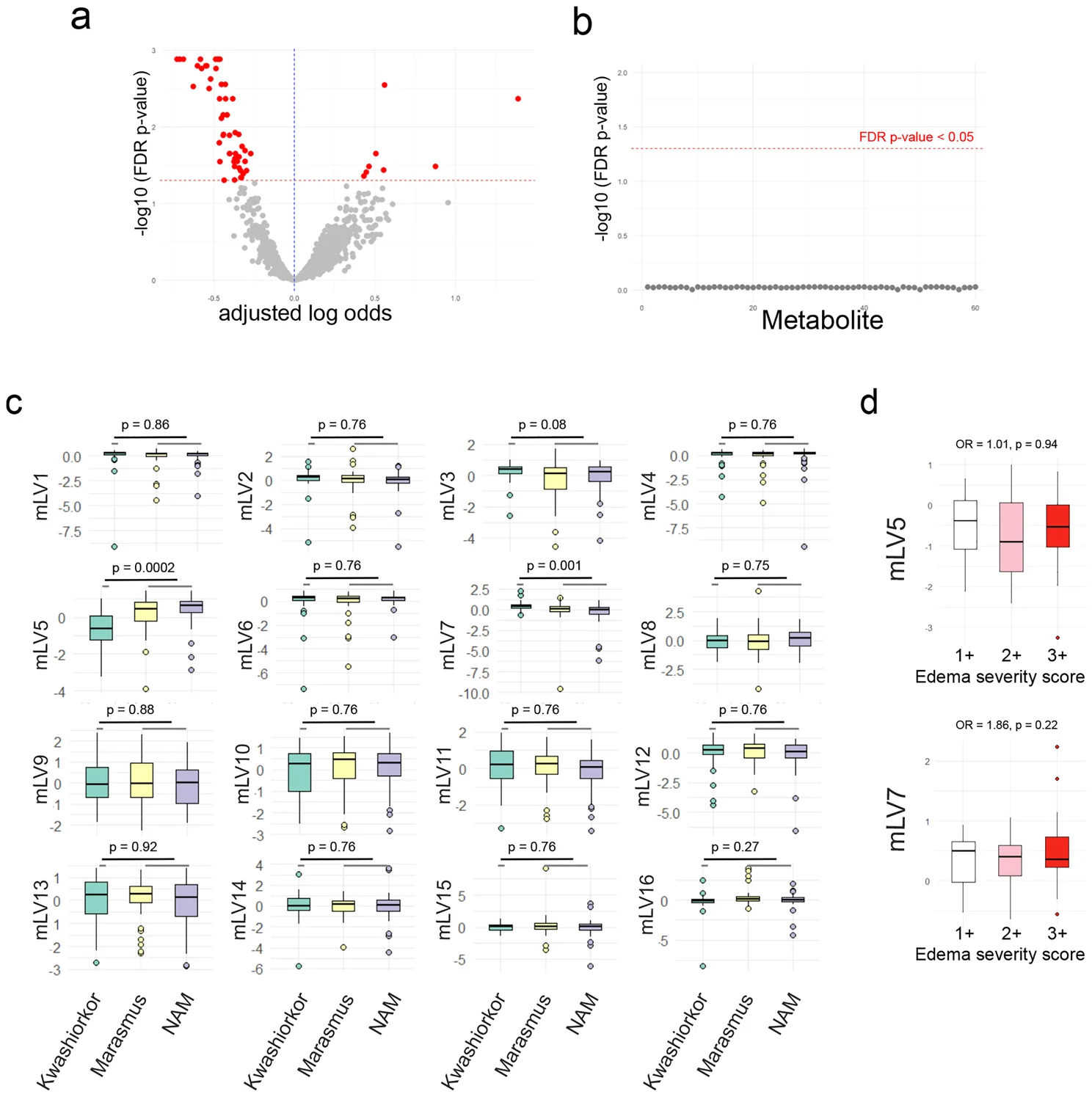
Untargeted semi-polar metabolomics analysis of plasma samples from children with kwashiorkor, marasmus and non-acutely malnourished (NAM) at baseline, discharge from hospital and treated (either end of nutritional rehabilitation for malnourished children or end of treatment for NAM). (**a**) Univariable analysis of metabolite features at baseline (*n* = 180). (**b**) Metabolite features showing difference at FDR *p* < 0.05 were tested for association with edema severity among children with kwashiorkor (*n* = 60). (**c**) Metabolome-related latent variables (mLV) associated with kwashiorkor, showing 2 out of 16 mLvs with difference at FDR *p* < 0.05 in kwashiorkor compared to marasmus and NAM combined (*n* = 180). (**d**) The 2 lLvs were tested for association with edema severity among children with kwashiorkor, where none were found to have an association (*n* = 60) (OR = odds ratio). Data for the individual metabolite features and mLvs across the different groups and time points can be visualized in this link: https://mudiboevans.Shinyapps.io/MSFKwash/

Plasma untargeted (semi-polar) metabolomics yielded a total of 2,825 features for both positive and negative ionization modes combined. Conditional logistic regression to account for the matched design on age, sex and triage score revealed 60 metabolite features different in kwashiorkor (FDR *p*-value < 0.05) compared to both marasmus and NAM combined (Fig. 3a). However, when tested for association with edema severity in kwashiorkor, none of these metabolite features were found to have passed criterion 2 (Fig. 3b).

Following FA, 16 metabolome-LVs (mLV) were extracted, of which only mLV5 and mLV7 passed criterion 1. Component metabolite features in each LV is shown in Supplementary file 1. Upon investigation of their association with kwashiorkor edema severity during nutritional recovery, we found no plasma mLV passing all 3 criteria, and hence no semi-polar metabolome-specific pathways were found to be associated with kwashiorkor in this study.

### Plasma multi-omics co-regulation

**Fig. 4:**
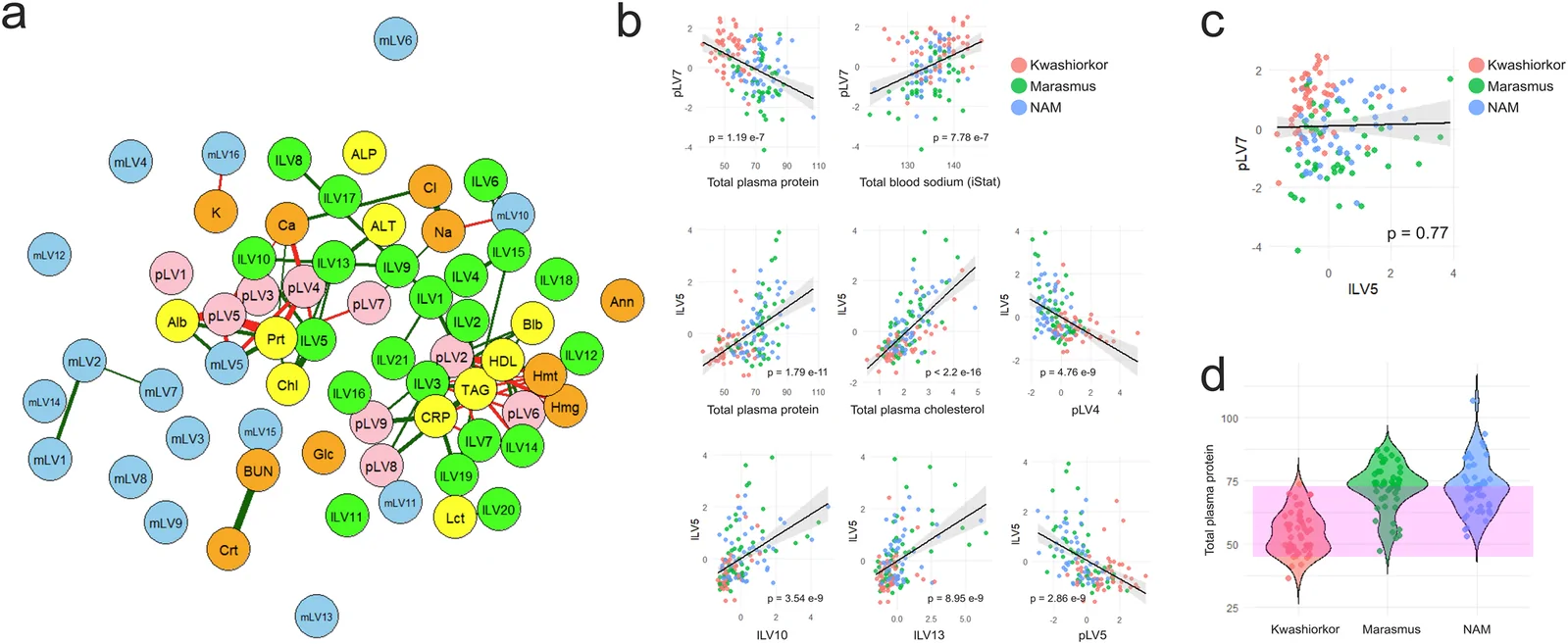
Multi-omics integration of plasma lipidome, (semi-polar) metabolome and proteome, and clinical biochemistry results. a. Partial correlation network analysis at baseline using a “spring” rendering (in *pcor* package in R). Plasma biochemical analyses: alb – albumin; ALP – alkaline phosphatase; ALT – alanine transaminase; blb – biliburin; chl – cholesterol; CRP – C-reactive protein; HDL – high density lipoprotein; prt – total protein; TAG – total triglyceride. Point-of-care analyses (iStat): ann – anion gap; BUN: blood urea nitrogen; Ca – ionized calcium; Cl – chloride; Crt – creatinine; glc – glucose; hmg – hemoglobin; hmt – hematrocrit; K – potassium; Na – sodium. b. Direct correlation among modules and clinical biochemistry parameters linked with pLV7 and lLV5. c. Direct correlation between pLV7 and lLV5. d. Plasma total protein concentration in the 3 groups (kwashiorkor, marasmus and NAM). The pink band represents children with the same total protein concentrations despite being in different groups

To integrate the multiple omics studies and to contextualize the clinical meaning of the individual latent variables (LV), we used partial correlation network analysis to determine patterns of association among all modules and clinical biochemistry results for all children (kwashiorkor, marasmus and NAM) at baseline (Fig. 4a). Plasma clinical biochemistry results are presented in Supplementary table 3. Nodes correspond to individual pLV, lLV, mLV or clinical biochemistry (both laboratory and point-of-care analysis) results for baseline plasma samples. Correlations are depicted by either a green line (indicating positive partial correlation) or a red line (negative partial correlation).

Linkages from the network (Fig. 4a) are isolated in Figure 4bto highlight the clinical and biochemical nodes associated with pLV7 and lLV5. As shown (Fig. 4b), pLV7 (which represents ECM proteins) was negatively associated with total plasma protein but positively associated with blood sodium concentration. On the other hand, lLV5 was positively associated with total plasma protein, total serum cholesterol, lLV10 and lLV13, and negatively associated with pLV4 and pLV5. lLV10 consists of 15 mostly phosphatidylethanolamine lipid species, whereas lLV13 is comprised of 5 unsaturated sphingomyelins. lLVs 10 and 13 were also found to be different in kwashiorkor compared to non-kwashiorkor, but their levels were not associated with kwashiorkor severity. pLV4 is composed of protein such as actin, complement component C7, protein virilizer homolog, angiotensinogen, carboxypeptidase N subunit 2, and hemoglobin subunit beta. pLV5 is composed of alpha-1-acid glycoprotein 1, complement C5, pigment epithelium-derived factor, leucine-rich alpha-2-glycoprotein, and zinc-alpha-2-glycoprotein. The composition of the different modules can be found in Supplementary file 1.

lLV5 and pLV7 are not directly correlated with each other (Fig. 4c) despite both being associated with total plasma protein. Consistent with most findings in the literature, including our previous studies, total plasma protein content is lower in kwashiorkor than in marasmus and NAM. However, there is a considerable overlap among the groups. Many children with marasmus and NAM had low total protein in their blood but did not manifest kwashiorkor syndrome. These associations remained robust in sensitivity analyses where children who tested positive for malaria in a rapid diagnostic test (Supplementary figure 5) were removed or that only children with kwashiorkor who tested negative for urine blood and protein using a urine test strip (Supplementary figure 6) were used for the analysis.

We acknowledge that partial correlation networks can reduce, but not eliminate, confounding by shared upstream processes such as inflammation, disease severity, or global nutritional status. Therefore, the observed associations should be interpreted as coordinated molecular signatures rather than direct biological interactions.

### Urinary metabolome and clinical biochemistry of children with kwashiorkor, marasmus and NAM

Due to difficulties in obtaining urine samples from children, we were only able to obtain baseline urine samples from 88 children (24 kwashiorkor, 32 marasmus, 32 NAM; patient characteristics in Supplementary table 4. Results for urinary biochemical analyses per group can be found in Supplementary table 5). Combining the positive and negative ionization modes together yielded a total of 4570 urine metabolomic features.

**Fig. 5:**
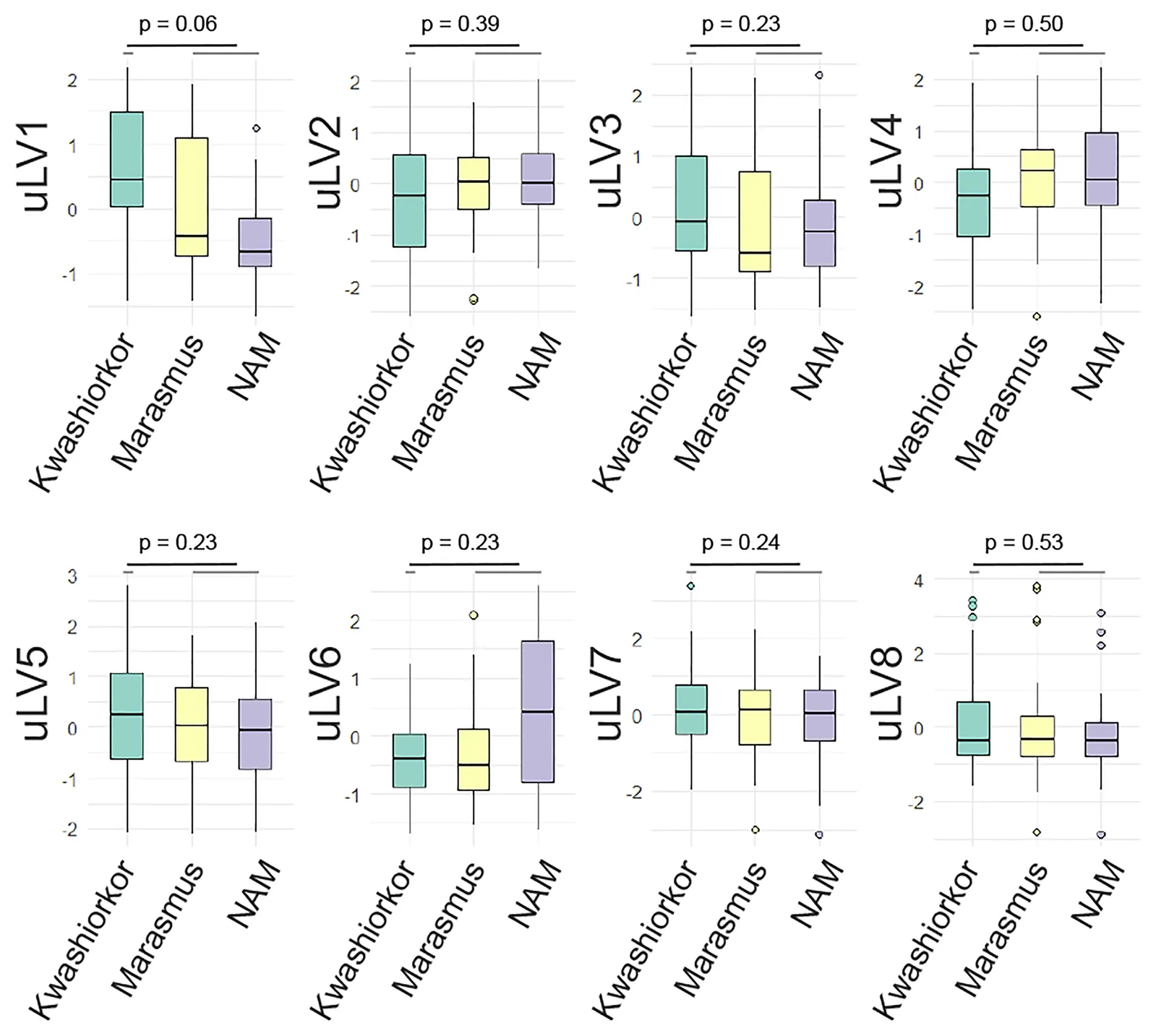
Untargeted semi-polar metabolomics analysis of urine samples from children with kwashiorkor (*n* = 24), marasmus (*n* = 32) and non-acutely malnourished (NAM) (*n* = 32) at baseline. Urine metabolome-related latent variables (uLV) associated with kwashiorkor, where no difference at FDR *p* < 0.05 in kwashiorkor compared to marasmus and NAM combined was found

After FA, the data was reduced to 8 urinary metabolome LVs (uLV). Since urine was only obtained at recruitment, only criteria 1 and 2 were applied to the urine results. There was no uLV associated with kwashiorkor (Fig. 5).

### Gut microbiome composition of children with kwashiorkor, marasmus and NAM

**Fig. 6:**
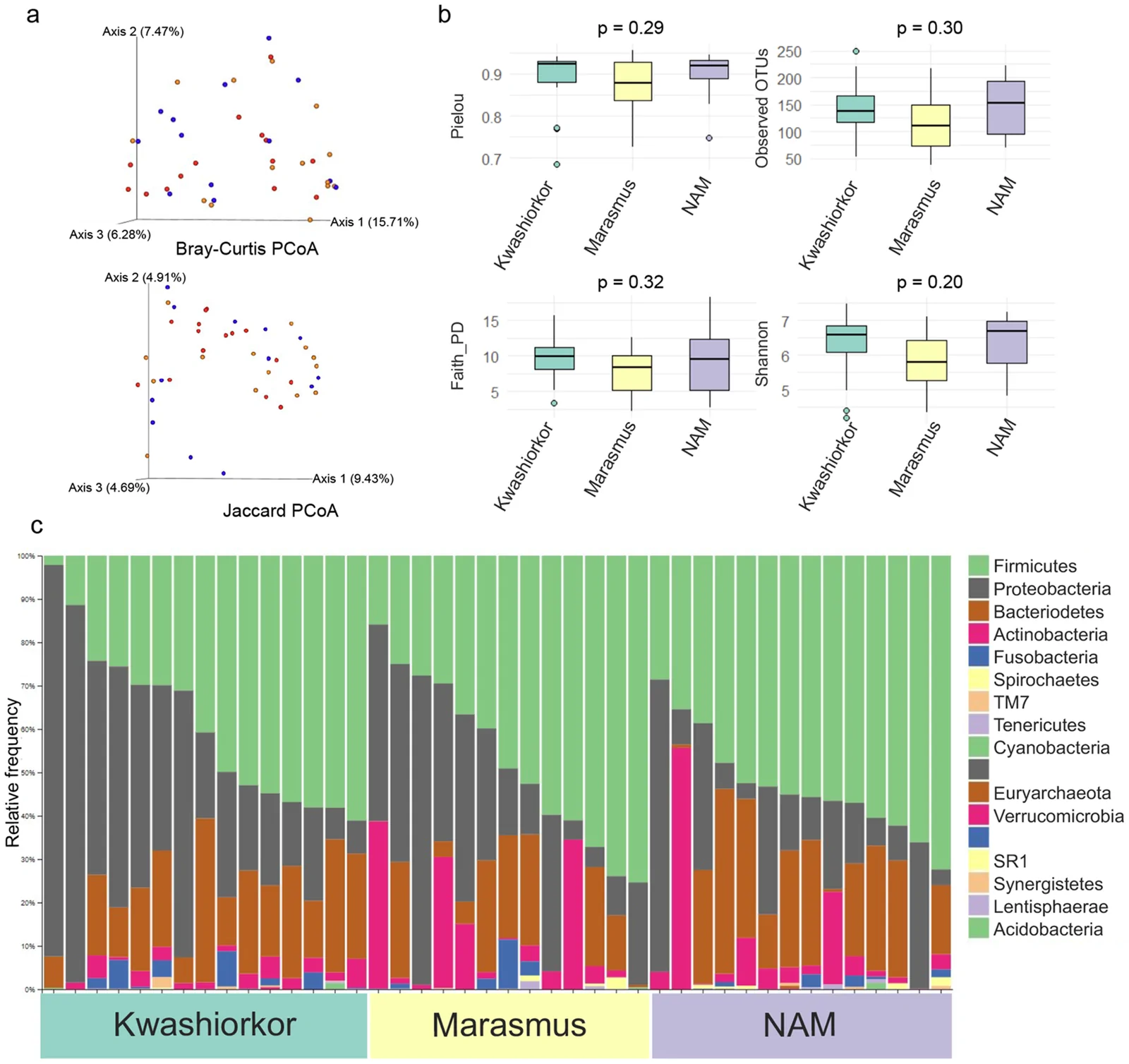
Gut microbiome differences between children with kwashiorkor (*n* = 14), marasmus (*n* = 13) and non-acutely malnourished children (*n* = 11). (**a**) Principal coordinates analysis using Bray-Curtis and Jaccard distances. (**b**) Comparison in alpha-diversity scores among the three groups. (**c**) Composition of gut microbiome in individuals with either kwashiorkor, marasmus or non-malnourished children

Out of 180 children at recruitment, we were only successfully able to collect fecal samples from 38 children (14 kwashiorkor, 13 marasmus, 11 NAM; patient characteristics in Supplementary table 6). Fecal samples were then subjected to 16s rRNA gene amplicon sequencing, and the resulting gene fragment amplicon sequences were quantified and taxonomically annotated. We initially visualized the variation in the data using principal coordinates analysis (PCoA) using Bray-Curtis and Jaccard distances. Using all distance measures, no clear clustering could be found neither generally nor on the basis of group (kwashiorkor vs marasmus vs NAM), age, sex and triage score (Fig. 6a). PERMANOVA analysis concurred with this visual inspection as differences among the groups were not significant. Moreover, no differences in alpha diversity metrics (Shannon, Faith Phylogenetic, Pielou) were found among the three groups (Fig. 6b). Finally, we used the ANCOM module in QIIME2 to identify differentially abundant features across sample groups at the taxonomic level of family, genus and strain. Both groupwise and pairwise analyses report no differentially abundant features (*p* > 0.05) among kwashiorkor, marasmus and NAM (Fig. 6c).

## Discussion

In this study, we identified ECM degradation and sphingolipid disruption as biomarkers of kwashiorkor, which may help further our understanding of its pathophysiology. Using a stringent multi-omics design, we uncovered latent variables that were distinct in kwashiorkor compared to marasmus and NAM children, correlated with oedema severity, and normalized with treatment. This approach delineates kwashiorkor-specific features that move beyond simple nutritional deficiency and instead reveal maladaptive tissue remodeling and lipid signaling.

We presented data from children with kwashiorkor, marasmus and NAM, matched based on age, sex, and a programmatic clinical triage score. Matching especially on triage score as a proxy for clinical severity upon admission was decided for the following reasons: first, NAM children without serious illness would overtly distinguish themselves from malnourished ill children only on the basis of illness severity. Second, certain diseases tend to co-occur with either form: for instance, HIV and malaria are reported to co-occur more with marasmus than kwashiorkor [43, 44]. Third, mortality rates have been reported to be higher in kwashiorkor than marasmus [45]. Other studies have reported the opposite, especially in areas with high burden of HIV [46]. Hence, matching for triage score, albeit imperfect, allowed to somehow disentangle pathways linked with kwashiorkor other than from clinical co-morbidities. However, differences remained between groups, with NAM children notably more likely to have a positive malaria rapid test.

This study further strengthens our previous findings on the role of ECM remodeling/degradation in the pathophysiology of kwashiorkor. We have previously shown that the protein lumican was higher in kwashiorkor compared to marasmus, was associated with severity of edema, and was resolving during treatment in Kenyan and Malawian children [16]. In the previous study, we matched the children with kwashiorkor and marasmus on the basis of serum albumin concentration, which led us to conclude that both hypoalbuminemia and heightened ECM degradation play important roles in kwashiorkor development. However, this previous work did not include NAM children, and only focused on proteomics.

In this current study, a pLV comprising lumican, gelsolin, and tetranectin met all biomarker criteria, reinforcing the role of ECM remodeling in kwashiorkor. Lumican, a small leucine-rich proteoglycan, regulates collagen fibrillogenesis and tissue integrity [47, 48]. This protein’s potential role in kwashiorkor has been described in our previous work [16]. Gelsolin, an actin-binding protein, also affects ECM by clearing actin filaments and modulating inflammation [49]. Plasma gelsolin (pGSN) plays key roles in actin scavenging, and immune modulation [49, 50]. pGSN levels have been reported to significantly decreased in tissue injury, secondary organ damage, and sepsis [51], but is increased in colon cancer, pancreatic cancer and pancreatitis [51], and HIV [52]. Tetranectin enhances plasminogen activation, important for fibrinolysis and ECM breakdown [53].

The increased circulating abundance of the pLV7 proteins may arise from enhanced synthesis or secretion, increased release from tissues during ECM remodelling or degradation, or altered clearance and redistribution. Previous protein-kinetic studies have demonstrated markedly reduced whole-body protein synthesis in children with kwashiorkor [54], making a generalized increase in protein synthesis a less likely explanation, although selective synthesis of individual proteins cannot be excluded. We therefore hypothesize that the circulating pLV7 signature most likely reflects increased release of ECM-related proteins during tissue remodelling or degradation. However, the present proteomic analysis cannot distinguish intact proteins from proteolytic fragments and cannot identify their tissue of origin. This interpretation should therefore be regarded as a biologically plausible hypothesis requiring confirmation through fragment-specific assays and tissue-level studies.

Nonetheless, using the human proteome atlas tool [42], we deduced that pLV7 represents a systemic ECM breakdown, as these proteins are co-expressed in a multitude of organs, including the lymphatic system and the liver. Our previous finding that plasma lymphatic vessel endothelial hyaluronan receptor 1 (LYVE1) levels are higher in kwashiorkor than marasmus, and are reduced after 60 days of post-discharge nutritional rehabilitation [16] indicating ECM degradation in the lymphatic system in kwashiorkor. Dysfunction of the lymphatic system integrity could potentially be playing a major role in edema formation in kwashiorkor, as it is the major route for interstitial fluid drainage back to circulation, as explained in the revised Starling model. The interstitial volume is mainly controlled by the activity of lymph flow which depends on the lymphatic function [55]. Damage to the lymph, i.e. degradation of lymph ECM, may contribute to edema formation. Moreover, ECM proteins are major constituents of the vessel wall supporting endothelial cells throughout the entire vascular system [56]. Degradation of the ECM in the vessel wall will therefore result in vascular leakage and increased capillary membrane permeability, further contributing to fluid leakage to the interstitium.

The ECM is also an important reservoir for sodium, enabled by the interaction between negatively charged ECMs, specifically glycosaminoglycans, allowing them to store non-osmotic Na^+^ ions. ECM is therefore an integral and dynamic component of sodium balance [57]. Our network analysis revealed a positive association between total blood sodium (via iStat point-of-care device) and pLV7. Disruption of the ECM integrity could potentially also influence sodium balance, and consequently contribute to water retention in kwashiorkor. Our results showed that children with kwashiorkor have higher blood sodium concentration compared to marasmus and NAM, which concur with previous findings [58]. However, it is worth noting that despite this statistical difference, median blood sodium levels in all children in this study still fall within normal range. No difference in urinary sodium was found between kwashiorkor and marasmus in this study, but both conditions had lower urinary sodium compared to NAM (Supplementary table 5).

The dysregulation of ECM homeostasis has also been implicated in liver pathologies, including liver fibrosis and non-alcoholic fatty liver disease [59]. It remains unclear whether systemic ECM homeostasis dysregulation is directly involved in kwashiorkor-related liver fat infiltration; but its participation in other liver pathologies makes this plausible.

We also found that lipid metabolism is strongly associated with kwashiorkor, particularly through latent lipid variable 5 (lLV5), which captures signatures of sphingomyelins, ceramides, and gangliosides. These lipids, collectively classified as sphingolipids, are key structural components of cell membranes, especially in the central nervous system, and play critical roles in cell signaling, proliferation, and apoptosis [60]. Ceramide serves as the core molecule, which can be modified to form sphingomyelins and further conjugated with glycolipids to produce gangliosides [61]. Thus, lLV5 likely reflects overall sphingolipid homeostasis. Since circulating sphingolipids are primarily synthesized by the liver and intestine, with only a small fraction derived directly from dietary sources [62], this may suggest that disturbances occurring in these organs may underlie the development of kwashiorkor. Moreover, network analysis showed that lLV5 is linked to total serum cholesterol, lLV10 (phosphatidylethanolamine lipid species), and lLV13 (unsaturated sphingomyelins), indicating that lLV5 may reflect a pathway involved in lipid transport, potentially in the liver. Our results however cannot support this hypothesis, which requires more in-depth investigation. Nonetheless, this offers a plausible next step for investigation.

Our findings can also be interpreted within the context of several historical and contemporary hypotheses proposed for the pathophysiology of kwashiorkor. While the present study was not designed to directly test these hypotheses, the identified signatures of ECM remodeling and sphingolipid homeostasis disruption provide a potential framework through which multiple upstream mechanisms may converge to produce the clinical phenotype.

Oxidative stress has long been considered a central driver of kwashiorkor [4]. Reactive oxygen species can directly promote ECM degradation through activation of matrix-remodeling enzymes and can disrupt membrane lipid composition [63], including sphingolipid metabolism. The concurrent elevation of ECM remodeling markers and depletion of sphingolipid-related lipids observed in our study is therefore compatible with a state of chronic oxidative injury, although our data cannot establish whether oxidative stress is the initiating event.

Similarly, immune activation and inflammation have frequently been implicated in kwashiorkor [5]. Both ECM remodeling and sphingolipid metabolism are tightly linked to inflammatory signaling. ECM degradation products can act as inflammatory mediators, while sphingolipids regulate immune cell trafficking, cytokine production, and vascular responses. The strong association of both signatures with edema severity and their normalization during recovery suggest that they may represent downstream manifestations of a sustained inflammatory process.

Our findings may also help contextualize previous observations of endothelial dysfunction in kwashiorkor. Maintenance of vascular barrier integrity depends on coordinated interactions between endothelial cells, ECM proteins [56], and membrane lipids [64]. Disturbances in ECM architecture together with altered sphingolipid homeostasis could contribute to increased vascular permeability and impaired fluid handling. Taken together, these findings further support the emerging view that edema in kwashiorkor cannot be explained solely by hypoalbuminemia, as we previously reported [16], but instead reflects broader disturbances in tissue structural integrity and lipid-mediated cellular homeostasis.

Our untargeted semi-polar metabolomics results did not reveal metabolites nor metabolome-LVs associated passing all kwashiorkor biomarker criteria. However, a targeted metabolomics study comparing kwashiorkor and marasmus revealed major differences in their metabolome, where 128 of 141 (91%) metabolites quantified were lower in serum of kwashiorkor than marasmus [65]. Another quantitative metabolite profiling study found reduced levels of metabolites involved in one-carbon metabolism in serum of kwashiorkor and marasmic-kwashiorkor compared to marasmus, especially levels of methionine and choline [15]. However, our previous targeted metabolomics study comparing kwashiorkor and marasmus matched for serum albumin concentrations showed an almost complete overlap of serum amino acids concentrations in both groups, except for phenylalanine which was lower in kwashiorkor [66]. Similar to our current study, a previous untargeted metabolomics study did not find semi-polar metabolomic differences between Nigerian children with kwashiorkor and marasmus [67]. It is possible that the metabolome is more susceptible to geographic differences, leading to inconsistent results from studies in different countries. Moreover, given the increased overall fluid retention in kwashiorkor, it is also possible that small hydrophilic metabolites are dispersed both in the intravascular volume as well as the interstitial space, which makes the apparent concentration of these metabolites lower in kwashiorkor. Our results do not suggest an increase in the intravascular space in kwashiorkor. While the endothelial glycocalyx serves as a barrier for proteins from crossing the endothelial space to the interstitial space, the movement of metabolites across the endothelium remains to be deeply investigated. Nonetheless, more investigation is needed to ascertain whether specific metabolite concentrations indeed play a role in kwashiorkor pathophysiology.

The role of the gut microbiome on the pathophysiology of kwashiorkor has previously been proposed. In a study of Malawian twins discordant for malnutrition, fecal transplantation of stool from children with kwashiorkor (*n* = 9) to germ-free mice led to phenotype of malnutrition where germ-free mice transplanted with stool from non-malnourished twin (*n* = 9) did not [6]. This study however did not include children with marasmus, and it is therefore difficult to determine whether the effect is due to kwashiorkor specifically or due to general malnutrition, or even clinical comorbidities. Also, edema in the mice models was not reported to have occurred. Similar to our findings, a previous study also did not find differences in gut microbiome between kwashiorkor and marasmus [67]. However, gut microbiome differences between children with kwashiorkor or marasmus and the NAM group cannot be excluded, given the small sample size, similar living conditions, and limited gastroenteritis cases. Our microbiome analysis was intended as an exploratory comparison only, and we acknowledge the limited power to detect taxonomic or functional differences. Future larger studies are therefore warranted.

This study has several limitations. First, we used factor analysis optimized for fast, matrix-free maximum-likelihood analysis for high-dimensional data, which may be prone to factor instability. We have nonetheless shown that the factors obtained in this study demonstrated modest to good stability. Second, we matched participants using a pragmatic triage score designed to identify children in need for immediate care. However, we acknowledge that this score, and hence matching for it, may not be optimal or detailed enough. Third, this study included only 60 children per group, which only offered an exploratory perspective to this complex problem. We have previously described the barriers and enablers in undertaking omics research in low-resource countries [68], such as Niger. Fourth, several logistic field operations may not be at par with quality standards observed in high income settings. Moreover, due to security reasons, we had to resort to training field staff remotely on certain aspects, such as collection of skin lesions data, which may have led to the low prevalence of detected cases. Fifth, we did not distinguish kwashiorkor from children presenting with both wasting and edema (marasmic-kwashiorkor). All children presenting with nutritional edema were considered kwashiorkor regardless of wasting. Sixth, we further acknowledge that our results were not validated using an independent cohort of children. Hence, research in other settings is needed to ensure reproducibility of the results. Nonetheless, the alignment of our current results with our previous work and that of others offers assurances on the validity of our findings. Finally, because factor analysis was performed separately within each omics layer and cross-omics relationships were subsequently assessed using partial correlations, our integration strategy does not explicitly model shared variation across omics platforms. Consequently, we cannot determine whether ECM remodeling and sphingolipid homeostasis dysregulation represent independent biological processes, sequential events, or parallel manifestations of a common upstream factor such as inflammation or disease severity. Future larger studies are needed to test shared and omics-specific sources of variation and to evaluate putative causal pathways.

## Conclusions

These observations suggest that severe malnutrition, and particularly kwashiorkor, is not simply a state of nutrient deficiency but a multifaceted physiological condition that reconfigures systemic molecular interactions. This results in increased ECM remodeling and sphingolipid homeostasis dysregulation, indicating that kwashiorkor could be linked to an atypical inflammatory response. Whether a specific inflammatory trigger is required to develop kwashiorkor or that children with kwashiorkor are predisposed to a derailed inflammatory response regardless of the stimuli remains to be investigated.

## Electronic supplementary material

Below is the link to the electronic supplementary material.

Supplementary material 1

Supplementary material 2

## Data Availability

Epicentre and MSF are committed to sharing and disseminating health data from its programs and research in an open, timely, and transparent manner to promote health for populations while respecting ethical and legal obligations towards patients, research participants, and their communities. For the purpose of this study, Epicentre and MSF collected identifiable participant data. Upon publication and for as long as the ethical authorisation permits it, the minimal data set underlying the findings of this study will be made available on request. If scientifically relevant, the request may be granted in accordance with legal framework set forth by MSF data sharing policy available on its website or on request. The MSF data sharing policy ensures that data will be available upon request to interested researchers while addressing all security, legal, and ethical concerns. All data access request for non-commercial and academic research can be addressed to epimail@epicentre.msf.org, parisrecherche@paris.msf.org or the corresponding authors. Such request will be submitted to the medical departments of MSF and Epicentre. In case of approval of the request, the data will be shared with researchers, subject to the establishment, within a reasonable timeline, of a data sharing agreement to provide the legal framework for data sharing – including any applicable data protection laws. Such data sharing agreement may differ depending on the nature of the data to be shared – pseudonymized or anonymized – and the sensitivity of the data. Analysis codes and summaries of metabolite, lipid, and protein features, and the gut microbiome analysis used in this study are publicly available at https://github.com/HOB-IDEALS/Kwash-Multi-omics. The data may be interactively visualized using this link: https://mudiboevans.shinyapps.io/MSFKwash/

## Abbreviations

ECM: Extracellular Matrix
ETAT: Emergency Triage Assessment and Treatment
FA: Factor Analysis
FDR: False Discovery Rate
lLV: Lipidome-related Latent Variable
LC-MS: Liquid chromatography-mass spectrometry
LV: Latent Variable
mLV: Metabolome-related Latent Variable
MUAC: Mid-Upper Arm Circumference
NAM: Non-Acutely Malnourished
pLV: Proteome-related Latent Variable
WHZ: Weight-for-Height Z-score
