# Supplementary material for: Multi-omics, multi-tissue analysis reveal role of extracellular matrix remodeling and sphingolipid homeostasis in edematous malnutrition (kwashiorkor)
Source: Biomark Res. 2026 Oct 28;14:151. doi: 10.1186/s40364-026-01016-9 (PMC13721215; doi:10.1186/s40364-026-01016-9)
Supplement: Supplementary file 1 [file 40364_2026_1016_MOESM2_ESM.docx]

SUPPLEMENTARY FIGURES AND TABLES

**Multi-omics, multi-tissue analysis reveal role of extracellular matrix remodeling and sphingolipid homeostasis in edematous malnutrition (kwashiorkor)**

Fabienne Nackers^1^†, Kirrily de Polnay^2^†, Ruben Almey^3^, Simon Daled^3^, Azahir Dan Djibo^4^, Sarah De Saeger^5^, Dieter Deforce^3^, Maarten Dhaenens^3^, Yannick Gansemans^3^, Griet Glorieux^6^, Ousmane Guindo^4^, Benjamin Jenkins^7,8^, Albert Koulman^7,8^, Hans-Joerg Lang^9,10^, Céline Langendorf^1^, Debby Laukens^11,12,13^, Christopher Mambula^14^, Isabelle Mukagatare^4^, Damon Parkington^7,8^, Elh Ibrahim Tassiou^15^, Wim Van Biesen^6^, Tom Van De Wiele^16^, Jill Van Massenhove^6^, Filip Van Nieuwerburgh^3^, Irène Adehossi^17^, André Briend^18,19^, Gerard Bryan Gonzales^20^*

^1^ Epicentre, 75019 Paris, France

^2^ Médecins Sans Frontières Operational Center of Brussels, Brussels, Belgium

^3^ Laboratory of Pharmaceutical Biotechnology, Faculty of Pharmaceutical Sciences, Ghent University, Ghent, Belgium

^4^ Epicentre, Maradi, Niger

^5^ Centre of Excellence in Mycotoxicology and Public Health, Department of Bioanalysis, Faculty of Pharmaceutical Sciences, Ghent University, Ghent, Belgium

^6^ Department of Internal Medicine and Paediatrics, Nephrology Section, Ghent University Hospital, Ghent, Belgium

^7^ Wellcome Trust - MRC Institute of Metabolic Science Metabolic Research Laboratories, Addenbrooke’s Hospital, Hills Road, Cambridge CB2 0QQ, UK

^8^ Medical Research Council Human Nutrition Research, Elsie Widdowson Laboratory, University of Cambridge, Fulbourn Road, Cambridge CB1 9NL, UK

^9^ Heidelberg Institute of Global Health, Heidelberg, Germany

^10^ NGO Alliance for International Medical Action/ALIMA, Dakar, Sénégal

^11^ Department of Internal Medicine and Pediatrics, Ghent University, Ghent, Belgium

^12^ VIB Center for Inflammation Research (IRC), Ghent University, Ghent, Belgium

^13^ Ghent Gut Inflammation Group (GGIG), Ghent University, Ghent, Belgium

^14^ Médecins Sans Frontières Operational Center of Paris, Paris, France

^15^ Health District Medical Officer, Madarounfa, Niger

^16^ Center for Microbial Ecology and Technology (CMET), Department of Biotechnology, Ghent University, Gent, Belgium

^17^ Niamey National Hospital, Niamey, Niger

^18^ Department of Nutrition, Exercise and Sports, Faculty of Science, University of Copenhagen, Frederiksberg, Denmark

^19^ Tampere Center for Child, Adolescent and Maternal Health Research, Faculty of Medicine and Health Technology, Tampere University and Tampere University Hospital, Tampere, Finland

^20^ Department of Public Health and Primary Care, Faculty of Medicine and Health Sciences, Ghent University, Ghent, Belgium

† equal contribution

Supplementary Table 1. Baseline characteristics of the study population

|  | | Kwashiorkor  (n=60) | Marasmus  (n=60) | No acute malnutrition (n=60) |
| --- | --- | --- | --- | --- |
| Age (months) | | **mean ± SD** | **mean ± SD** | **mean ± SD** |
|  | | 24.7 (7.8) | 21.3 (9.0) | 25.0 (12.1) |
| Age (categories) | | **n (%)** | **n (%)** | **n (%)** |
| 6-11 | | 2 (3.3) | 11 (18.3) | 6 (10.0) |
| 12-23 | | 27 (45.0) | 20 (33.3) | 24 (40.0) |
| 24-35 | | 24 (40.0) | 26 (43.3) | 17 (28.3) |
| 36-47 | | 5 (8.3) | 2 (3.3) | 8 (13.3) |
| 48-59 | | 2 (3.3) | 1 (1.7) | 5 (8.3) |
| Sex | | **n (%)** | **n (%)** | **n (%)** |
| Female | | 36 (60.0) | 36 (60.0) | 36 (60.0) |
| Triage score | | **n (%)** | **n (%)** | **n (%)** |
| Green (non-urgent) | | 30 (50.0) | 30 (50.0) | 30 (50.0) |
| Yellow (priority) | | 29 (48.3) | 29 (48.3) | 29 (48.3) |
| Red (emergency) | | 1 (1.7) | 1 (1.7) | 1 (1.7) |
| PEWS at baseline | | **n (%)** | **n (%)** | **n (%)** |
| Cut-off | |  |  |  |
| 0-2 (green) | | 58 (96.7) | 57 (95.0) | 46 (76.7) |
| 3-4 (yellow) | | 2 (3.3) | 1 (1.7) | 11 (18.3) |
| 5-6 (orange) | | 0 (00.0) | 0 (00.0) | 2 (3.3) |
| ≥ 7 (red) | | 0 (00.0) | 2 (3.3) | 1 (1.7) |
| Respiratory rate | | **n (%)** | **n (%)** | **n (%)** |
| 6-11 months | 12-59 months |  |  |  |
| >70 | >60 | 0 (00.0) | 0 (00.0) | 1 (1.7) |
| 51-70 | 41-60 | 6 (10.5) | 5 (8.8) | 22 (36.7) |
| 30-50 | 25-40 | 51 (89.5) | 52 (91.2) | 37 (61.7) |
| <30 | <25 | 0 (00.0) | 0 (00.0) | 0 (00.0) |
| missing values | | 3 | 3 | 0 |
| Signs of respiratory distress | | **n (%)** | **n (%)** | **n (%)** |
| Yes | | 1 (1.7) | 2 (3.4) | 4 (6.7) |
| No | | 59 (98.3) | 57 (96.6) | 56 (93.3) |
| missing values | | 0 | 1 | 0 |
| O2 saturation% (ambient air) | | **n (%)** | **n (%)** | **n (%)** |
| ≥ 93 | | 58 (98.3) | 58 (98.3) | 59 (98.3) |
| < 93 | | 1 (1.7) | 1 (1.7) | 1 (1.7) |
| missing values | | 1 | 1 | 0 |
| Heart rate | | **n (%)** | **n (%)** | **n (%)** |
| 6-11 months | 12-59 months |  |  |  |
| >190 | >170 | 0 (00.0) | 2 (3.5) | 3 (5.0) |
| 181-190 | 161-170 | 1 (1.7) | 1 (1.7) | 6 (10.0) |
| 161-180 | 141-160 | 5 (8.6) | 9 (15.5) | 13 (21.7) |
| 90-160 | 80-140 | 52 (89.7) | 46 (79.3) | 38 (63.3) |
| <90 | <80 | 0 (00.0) | 0 (00.0) | 0 (00.0) |
| missing values | | 2 | 2 | 0 |
| Capillary refill | | **n (%)** | **n (%)** | **n (%)** |
| >3 sec | | 0 (00.0) | 0 (00.0) | 0 (00.0) |
| ≤3 sec | | 59 (100.0) | 60 (100.0) | 60 (100.0) |
| missing values | | 1 | 0 | 0 |
| Level of consciousness | | **n (%)** | **n (%)** | **n (%)** |
| unresponsive | | 0 (00.0) | 0 (00.0) | 0 (00.0) |
| pain | | 0 (00.0) | 0 (00.0) | 0 (00.0) |
| verbal | | 3 (5.1) | 2 (3.3) | 5 (8.3) |
| alert | | 56 (94.9) | 58 (96.7) | 55 (91.7) |
| missing values | | 1 | 0 | 0 |
| Temperature (° C) | | **n (%)** | **n (%)** | **n (%)** |
| ≥ 38 | | 3 (5.0) | 8 (13.6) | 18 (30.0) |
| 36.0 - 37.9 | | 57 (95.0) | 49 (83.1) | 42 (70.0) |
| ≤ 35.9 | | 0 (00.0) | 2 (3.4) | 0 (00.0) |
| missing values | | 0 | 1 | 0 |
| Level of edema at baseline | | **n (%)** | **n (%)** | **n (%)** |
| 0 | | / | 60 (100.0) | 60 (100.0) |
| + | | 21 (35.0) | / | / |
| ++ | | 19 (31.7) | / | / |
| +++ | | 20 (33.3) | / | / |
| Middle upper arm circumference | |  |  |  |
| Median | | 122.5 | 111.0 | 139.0 |
| IQR [25,75] | | [115 , 130] | [107 , 116.5] | [133 , 145.5] |
| MUAC categories | | **n (%)** | **n (%)** | **n (%)** |
| ≥125 mm | | 28 (46.7) | 5 (8.3)* | 60 (100.0) |
| 115-124 mm | | 19 (31.7) | 15 (25.0)* | 0 (00.0) |
| <115 mm | | 13 (21.7) | 40 (66.7) | 0 (00.0) |
| Weight for height z-score | |  |  |  |
| Median | | -2.14 | -3.61 | -1.26 |
| IQR [25,75] | | [-2.84 , -1.04] | [-4.23 , -3.20] | [-1.60 , -0.77] |
| Weight for height z-score categories | | **n (%)** | **n (%)** | **n (%)** |
| WHZ ≥ -2 | | 29 (48.3) | 0 (0.00) | 60 (100.0) |
| -3 ≤ WHZ < -2 | | 18 (30.0) | 6 (10.0) | 0 (00.0) |
| WHZ < -3 | | 13 (21.7) | 54 (90.0) | 0 (00.0) |
| Weight for age z-score | |  |  |  |
| Median | | -3.25 | -4.16 | -1.81 |
| IQR [25,75] | | [-4.11 , -1.94] | [-4.91 , -3.58] | [-2.36 , -1.03] |
| Weight for age z-score categories | | **n (%)** | **n (%)** | **n (%)** |
| WAZ ≥ -2 | | 17 (28.3) | 0 (0.00) | 35 (58.3) |
| -3 ≤ WAZ < -2 | | 12 (20.0) | 7 (11.7) | 23 (38.3) |
| WAZ < -3 | | 31 (51.7) | 53 (88.3) | 2 (3.33) |
| Height for age z-score | |  |  |  |
| Median | | -3.29 | -2.88 | -1.79 |
| IQR [25,75] | | [-4.09 , -2.06] | [-4.59 , -1.95] | [-2.36 , -0.71] |
| Height for age z-score categories | | **n (%)** | **n (%)** | **n (%)** |
| HAZ ≥ -2 | | 13 (21.7) | 15 (25.0) | 35 (58.3) |
| -3 ≤ HAZ < -2 | | 11 (18.3) | 16 (26.7) | 18 (30.0) |
| HAZ < -3 | | 36 (60.0) | 29 (48.3) | 7 (11.7) |
| Height | | **mean ± SD** | **mean ± SD** | **mean ± SD** |
| Centimeters | | 76.6 ± 6.6 | 73.5 ± 5.6 | 81.1 ± 9.1 |
| Weight | | **mean ± SD** | **mean ± SD** | **mean ± SD** |
| Kilograms | | 8.2 ± 1.9 | 6.5 ± 1.0 | 9.7 ± 2.1 |
| Signs and symptoms  (can be multiple per child) | | **n (%)** | **n (%)** | **n (%)** |
| Clinical anemia, severe | | 1 (1.7) | 4 (6.7) | 14 (23.3) |
| Clinical anemia, moderate | | 8 (13.3) | 7 (11.7) | 10 (16.7) |
| No clinical anemia | | 51 (85.0) | 49 (81.7) | 36 (60.0) |
| Dehydration, severe | | 0 (00.0) | 1 (1.7) | 1 (1.7) |
| Dehydration, moderate | | 2 (3.4) | 6 (10.0) | 1 (1.7) |
| No dehydration | | 57 (96.6) | 53 (88.3) | 58 (95.7) |
| Missing values | | 1 | 0 | 0 |
| Diarrhea (≥3 stools) | | 16 (26.7) | 35 (58.3) | 14 (23.3) |
| No diarrhea (<3 stools) | | 44 (73.3) | 25 (41.7) | 46 (76.7) |
| Cough | | 12 (20.0) | 21 (35.0) | 21 (35.6) |
| No cough | | 48 (80.0) | 39 (65.0) | 38 (64.4) |
| Missing values | | 0 | 0 | 1 |
| Upper respiratory tract infection | | 6 (10.0) | 14 (23.3) | 17 (28.3) |
| No upper resp. infection | | 54 (90.0) | 46 (76.7) | 43 (71.7) |
| Lower respiratory tract infection | | 7 (11.7) | 6 (10.0) | 7 (11.7) |
| No lower resp. infection | | 53 (88.3) | 54 (90.0) | 53 (88.3) |
| Otitis | | 0 (00.0) | 0 (00.0) | 2 (3.3) |
| No otitis | | 60 (100.0) | 60 (100.0) | 58 (77.8) |
| Hepatomegaly | | 0 (00.0) | 0 (00.0) | 1 (1.7) |
| No hepatomegaly | | 60 (100.0) | 60 (100.0) | 49 (98.3) |
| Splenomegaly | | 0 (00.0) | 0 (00.0) | 1 (1.7) |
| No splenomegaly | | 60 (100.0) | 60 (100.0) | 59 (98.3) |
| Edema | | **n (%)** | **n (%)** | **n (%)** |
| Feet (+) | | 60 (100.0) | / | / |
| Ankle (+) | | 55 (91.7) | / | / |
| Legs (++) | | 39 (65.0) | / | / |
| Face (+++) | | 20 (33.3) | / | / |
| Hands (+++) | | 19 (31.7) | / | / |
| Forearms (+++) | | 17 (28.3) | / | / |
| Arms (+++) | | 17 (28.3) | / | / |
| Trunk (+++) | | 2 (3.3) | / | / |
| Perineum (+++) | | 2 (3.3) | / | / |
| Median days (min-max) since caregiver saw edema starts | |  | / | / |
| + | | 2 [3-15] |  |  |
| ++ | | 3 [1-14] | / | / |
| +++ | | 4 [2-14] | / | / |
| Measles vaccination | | **n (%)** | **n (%)** | **n (%)** |
| Yes (card or verbal) | | 41 (68.3) | 51 (85.0) | 53 (88.3) |
| No | | 17 (28.3) | 5 (8.3) | 7 (11.7) |
| Unknown | | 2 (3.3) | 4 (6.7) | 0 (00.0) |
| Ever admitted to the ATFC before this episode | | |  |  |
| No | | 42 (70.0) | 34 (56.7) | 58 (98.3) |
| Yes, once | | 16 (26.7) | 20 (33.3) | 1 (1.7) |
| Yes, more than once | | 2 (3.3) | 6 (10.0) | 0 (0.0) |
| Unknown | | 0 | 0 | 1 |
| Ever admitted to the ITFC before this episode | | |  |  |
| No | | 57 (95.0) | 55 (91.7) | 59 (98.3) |
| Yes, once | | 2 (3.3) | 4 (6.7) | 1 (1.7) |
| Yes, More than once | | 1 (1.7) | 1 (1.7) | 0 (0.0) |
| Ever had kwashiorkor before this episode | | 7 (11.7) | 0 (exclusion criteria) | |
| History of kwashiorkor among siblings | | 8 (13.3) | 3 (5.1) | 4 (6.7) |
| Education level (head of household) | | **n (%)** | **n (%)** | **n (%)** |
| No formal education | | 11 (20.4) | 11 (20.4) | 17 (33.3) |
| Koranic school | | 33 (61.1) | 32 (59.3) | 27 (52.9) |
| Primary school (completed or not) | | 2 (3.7) | 5 (9.3) | 2 (3.9) |
| CEG (completed or not) | | 5 (9.3) | 5 (9.3) | 5 (9.8) |
| Other education form | | 3 (5.7) | 1 (1.9) | 0 (00.0) |
| Unknown | | 6 | 6 | 9 |
| Water source | | **n (%)** | **n (%)** | **n (%)** |
| Tap/running water | | 46 (76.7) | 37 (61.7) | 40 (66.7) |
| Borehole/pump | | 5 (8.3) | 9 (15.0) | 6 (10.0) |
| Protected well | | 3 (5.0) | 5 (8.3) | 2 (3.3) |
| Unprotected well | | 3 (5.0) | 7 (11.7) | 9 (15.0) |
| Surface water | | 3 (5.0) | 2 (3.3) | 2 (3.3) |
| Other water source | | 0 (00.0) | 0 (00.0) | 1 (1.7) |
| Sanitation | | **n (%)** | **n (%)** | **n (%)** |
| Flush toilets | | 0 (0.0) | 1 (1.7) | 0 (0.0) |
| Latrines | | 32 (53.3) | 39 (65.0) | 42 (70.0) |
| No sanitation facilities | | 28 (46.7) | 20 (33.3) | 17 (28.3) |
| Do not wish to answer | | 0 (00.0) | 0 (00.0) | 1 (1.7) |

PEWS; Paediatric Early Warning Score

*Children with WHZ-scores <-3

Supplementary Table 2. Study triage score, adapted from the Emergency triage assessment and treatment (ETAT) applied in the MSF program in Niger at the time of the study

| Triage score | Signs | |
| --- | --- | --- |
| Emergency  (Need immediate emergency treatment)  Red | Obstructed or absent breathing  Central Cyanosis  Severe respiratory distress  Signs of shock: cold extremities with capillary refill >3 seconds and weak and fast pulse  Coma (AVPU* assessment)  Convulsing/seizures (now)  Severe dehydration (Diarrhoea plus any two of lethargy or unconsciousness, sunken eyes, very slow return of skin after pinching) | |
| Priority  (Need assessment and rapid attention)  Yellow | Temperature very high  Severe pallor  Severe pain  Respiratory distress  Restless, continuously irritable, or lethargic  Referral (urgent) | *Not considered for the study/matching*  *Tiny baby, infant (any sick child<2months)*  *Trauma or other urgent surgical condition*  *History of poisoning*  *Burns (major)*  *Malnutrition: visible severe wasting*  *Edema of both feet* |
| Non-urgent  (Can queue)  Green | Absence of urgent or priority criteria - This category might include children with severe acute malnutrition hospitalized because they failed at the appetite test but have no other medical reason for being hospitalized | |

*A : Alert, V : react to voice, P : to pain, U: Unresponsive

**Remark:** A consistent lack of appetite is a sign of major complication in severe acute malnutrition. Indeed, children with severe acute malnutrition with infections may show no signs or symptoms but lack of appetite. Failing an appetite test (with RUTF) is a criterion for immediate inpatient care. However, the appetite will not be considered in the score for matching as the appetite test is not routinely done in non-malnourished children, making comparison (and thus matching) difficult.

Categories were relatively broad to limit the impact of matching on time for enrolment as well as to avoid risking overmatching.

In case a child deteriorates more than 24 hours after admission (with possible transfer from the ATFC to the ITFC or from a non-intensive inpatient unit to the Intensive Care Unit, ICU), the child remained classified and matched according to his/her triage score at baseline for the primary analysis.

Supplementary Table 3: Plasma biochemical analysis results

|  | Kwashiorkor  (n=60) | Marasmus  (n=60) | NAM (n=60) |
| --- | --- | --- | --- |
|  |  |  |  |
| Triglycerides (mmol/L) |  |  |  |
| Median [min, max] | 1.27 [0.45,3.39] | 1.51 [0.49,5.75] | 1.83 [0.45,5.18] |
| *Missing values* | 3 | 3 | 2 |
| Total cholesterol (mmol/L) |  |  |  |
| Median [min, max] | 1.71 [0.52,4.36] | 2.11 [1.12,4.18] | 2.11 [1.3,4.89] |
| *Missing values* | 3 | 3 | 2 |
| HDL cholesterol (mmol/L) |  |  |  |
| Median [min, max] | 0.31 [0.20,1.22] | 0.34 [0.20,1.54] | 0.27 [0.20,1.39] |
| *Missing values* | 3 | 3 | 2 |
| Calculated LDL cholesterol (mmol/L) |  |  |  |
| Median [min, max] | 0.70 [0.01,3.00] | 0.90 [0.01,3.00] | 0.90 [0.01,3.20] |
| *Missing values* | 3 | 6 | 2 |
| ALP (U/L) |  |  |  |
| Median [min, max] | 140 [0.01,382] | 152 [72,375] | 187.5 [0.01,309] |
| *Missing values* | 3 | 3 | 2 |
| ALT (U/L) |  |  |  |
| Median [min, max] | 35 [0.01,1179] | 18.5 [0.01,156] | 20 [7,382] |
| < 15 | 6 (10.5) | 16 (28.6) | 18 (31.6) |
| 15 – 39 | 28 (49.1) | 34 (60.7) | 33 (57.9) |
| 40 – 79 | 12 (21.1) | 4 (7.1) | 4 (7.0) |
| ≥ 80 | 11 (19.3) | 2 (3.6) | 2 (3.5) |
| *Missing values* | 3 | 4 | 3 |
| AST (U/L) |  |  |  |
| Median [min, max] | 49 [25,285] | 46 [21,186] | 44 [20,342] |
| *Missing values* | 7 | 7 | 10 |
| CRP (mg/L) |  |  |  |
| Median [min, max] | 13.1 [0.01,242] | 12.4 [0.01,274] | 72.9 [0.01,258] |
| < 1 | 14 (24.6) | 12 (21.1) | 9 (15.5) |
| 1 – < 5 | 8 (14.0) | 5 (8.8) | 2 (3.5) |
| 5 – < 15 | 8 (14.0) | 13 (22.8) | 3 (5.2) |
| ≥ 15 | 27 (47.4) | 27 (47.4) | 44 (75.9) |
| *Missing values* | 3 | 3 | 2 |
| Total bilirubin (mg/dL) |  |  |  |
| Median [min, max] | 0.3 [0.1,6.6] | 0.3 [0.01,2.7] | 0.4 [0.1,3.0] |
| < 0.5 | 44 (77.2) | 43 (75.4) | 30 (51.7) |
| 0.5 – <2.0 | 11 (19.3) | 13 (22.8) | 24 (41.4) |
| ≥ 2.0 | 2 (3.5) | 1 (1.8) | 4 (6.9) |
| *Missing values* | 3 | 3 | 2 |
| Albumin (g/L) |  |  |  |
| Median [min, max] | 17.3 [<9.0,32.5] | 29.9 [<9.0,50.3] | 31.9 [<9.0,42.6] |
| < 25 | 50 (87.7) | 20 (35.1) | 13 (22.4) |
| 25 – 34 | 7 (12.3) | 23 (40.4) | 27 (46.6) |
| 35 – 39 | 0 | 6 (10.5) | 13 (22.4) |
| ≥ 40 | 0 | 8 (14.0) | 5 (8.6) |
| *Missing values* | 3 | 3 | 2 |
| Total protein (g/L) |  |  |  |
| Median [min, max] | 53.2[36.5,73.6] | 73.7 [47.1,91.9] | 72.0 [52.8,106.6] |
| *Missing values* | 3 | 3 | 2 |
| Malaria rapid test | **n (%)** | **n (%)** | **n (%)** |
| *Positive* | 32 (53.3) | 29 (48.3) | 41 (73.2) |
| *Negative* | 28 (46.7) | 31 (51.7) | 15 (26.8) |
| *Not tested* | 0 | 0 | 4 |
| Urine strip | **n (%)** | **n (%)** | **n (%)** |
| Proteins [mg/dl]  Negative  Trace  30  100  500  Missing values | 22 (75.9)  7 (24.1)  0 (00.0)  0 (00.0)  0 (00.0)  31 | 12 (44.4)  9 (33.3)  3 (11.1)  2 (7.4)  1 (3.7)  33 | 13 (48.2)  11 (40.7)  3 (11.1)  0 (00.0)  0 (00.0)  33 |
| Blood [Erythrocytes/µl]  Negative  + [5-10]  ++ [50]  +++ [300]  Missing value | 23 (82.1)  2 (7.4)*  3 (10.7)*  0 (00.0)  32 | 22 (81.5)  2 (7.4)  1 (3.7)  2 (7.4)  33 | 24 (88.9)  2 (7.4)  1 (3.7)  0 (00.0)  33 |
| Point-of-care analysis - iSTAT |  |  |  |
| Time from sampling to iSTAT analysis |  |  |  |
| < 30 min | 38 (63.3) | 48 (80.0) | 57 (95.0) |
| ≥ 30 min | 22 (36.7) | 12 (20.0) | 3 (5.0) |
| Sodium [Na] mmol/L |  |  |  |
| Median [min, max] | 137 [125,146] | 135 [120,142] | 134 [123,143] |
| < 125 | 0 (00.0) | 2 (3.6) | 1 (1.7) |
| 125 – 134 | 19 (31.7) | 25 (45.5) | 30 (50.0) |
| 135 – 144 | 40 (66.7) | 28 (50.9) | 29 (48.3) |
| ≥ 145 | 1 (1.7) | 0 (00.0) | 0 (00.0) |
| *Missing values* | 0 | 5 | 0 |
| Ionized Calcium [iCa] mmol/L |  |  |  |
| Median [min, max] | 1.15 [0.75,1.28] | 1.18 [0.95,1.45] | 1.18 [1.03,1.35] |
| *Missing values* | 1 | 5 | 1 |
| Potassium [K] mmol/L |  |  |  |
| Median [min, max] | 3.1 [2,9] | 3.4 [2,5.3] | 3.8 [2.2,5.3] |
| < 2.5 | 9 (16.1) | 8 (15.4) | 1 (1.7) |
| 2.5-< 3.5 | 25 (44.6) | 18 (34.6) | 18 (31.0) |
| 3.5 – <4.0 | 12 (21.4) | 12 (23.1) | 15 (25.9) |
| 4.0 – <5.0 | 6 (10.7) | 12 (23.1) | 22 (37.9) |
| ≥ 5.0 | 4 (7.1) | 2 (3.9) | 2 (3.5) |
| *Missing values* | 4 | 8 | 2 |
| Chloride [Cl] mmol/L |  |  |  |
| Median [min, max] | 104 [84,116] | 101 [80,117] | 102 [79,111] |
| *Missing values* | 0 | 5 | 1 |
| Urea Nitrogen [BUN] mg/dL |  |  |  |
| Median [min, max] | <0.03 [<0.03,98] ** | 4 [<0.03,30] | 6 [<0.03,36] |
| *Missing values* | 0 | 5 | 0 |
| Urea Nitrogen [BUN] mg/dL excluding outlier (BUN=98) |  |  |  |
| Median [min, max] | <0.03 [<0.03, 13] | 4 [<0.03,30] | 6 [<0.03,36] |
| *Missing values* | 1 | 5 | 0 |
| Creatinine mg/dL |  |  |  |
| Median [min, max] | <0.2 [<0.2,6.4] | <0.2 [<0.2,6] | <0.2 [<0.2,0.4] |
| < 0.3 | 52 (88.1) | 46 (85.2) | 56 (93.3) |
| 0.3 – 0.4 | 5 (8.5) | 5 (9.3) | 4 (6.7) |
| – 0.9 | 1 (1.7) | 1 (1.9) | 0 |
| 1.0 - 3.9 | 0 | 0 | 0 |
| ≥ 4 | 1 (1.7)** | 2 (3.7) | 0 |
| *Missing values* | 1 | 6 | 0 |
| Haemoglobin [Hb] g/dL |  |  |  |
| Median [min, max] | 9.2 [4.7,12.9] | 9.9 [4,15] | 9.2 [2.3,12.9] |
| < 7 | 0 | 2 (3.6) | 1 (1.7) |
| 7.0 – 9.9 | 12 (20.0) | 7 (12.3) | 18 (30.0) |
| 10.0 – 10.9 | 27 (45.0) | 22 (38.6) | 22 (36.7) |
| 11.0 – 11.9 | 10 (16.7) | 9 (15.8) | 7 (11.7) |
| ≥ 12 | 11 (18.3) | 19 (33.3) | 13 (21.7) |
| *Missing values* | 0 | 3 | 0 |
| Haematocrit [Hct]% |  |  |  |
| Median [min, max] | 27 [15,38] | 30 [<15,44] | 27 [<15,38] |
| < 21 | 12 (20.0) | 5 (9.1) | 18 (30.0) |
| 21 – 29 | 27 (45.0) | 22 (40.0) | 22 (36.7) |
| 30 – 32 | 10 (16.7) | 9 (16.4) | 7 (11.7) |
| ≥ 33 | 11 (18.3) | 19 (34.6) | 13 (21.7) |
| *Missing values* | 0 | 5 | 0 |
| Glucose [Glu] mg/dL |  |  |  |
| Median [min, max] | 79 [38,118] | 88.5 [47,163] | 91.5 [51,158] |
| < 70 | 17 (28.8) | 5 (9.4) | 4 (6.7) |
| 70 – 89 | 30 (50.9) | 25 (47.2) | 25 (41.7) |
| 90 – 119 | 12 (20.3) | 19 (35.9) | 23 (38.3) |
| 120-160 | 0 | 4 (7.6) | 8 (13.3) |
| *Missing values* | 1 | 7 | 0 |
| Lactate [Lac] mmol/L |  |  |  |
| Median [min, max] | 2.2 [0.6,5.3] | 1.8 [0.6,11.3] | 1.9 [0.7,9.3] |
| < 2.0 | 26 (45.6) | 32 (55.2) | 38 (63.3) |
| 2.0 – < 3.0 | 14 (24.6) | 17 (29.3) | 6 (10.0) |
| 3.0 – <5.0 | 16 (28.1) | 7 (12.1) | 14 (23.3) |
| ≥ 5 | 1 (1.8) | 2 (3.5) | 2 (3.3) |
| *Missing values* | 3 | 2 | 0 |

* 2 invalid tests because urines were contaminated by stools; 3 results diagnosed as urinary tract infection by a pediatrician.

**Creatinine of 6.4 mg/dL and urea nitrogen of 98 mg/dL in a child who presented neither proteins nor blood in urine strip.

Supplementary Table 4. Characteristics of the subset of participants with urine samples

|  | Kwashiorkor  (n=24) | Marasmus  (n=32) | No acute malnutrition (NAM) (n=32) |
| --- | --- | --- | --- |
| Age (months) mean ± SD | 22 ± 6.1 | 21.2 ± 9.1 | 25.5 ± 12.9 |
| Sex (n female, %) | 9 (37) | 19 (59) | 16 (50) |
| Triage score* n (%) |  |  |  |
| Green (non-urgent) | 9 (38) | 15 (47) | 14 (44) |
| Yellow (priority) | 14 (58) | 16 (50) | 17 (53) |
| Red (emergency) | 1 (4) | 1 (3) | 1 (3) |
| Initial recruitment site n (%) |  |  |  |
| Children hospitalized | 15 (63) | 17 (53) | 18 (56) |
| Children in outpatient care | 9 (37) | 15 (47) | 14 (44) |
| Level of edema at baseline  n (%) |  |  |  |
| 0 | 0 (0) | 32 (100) | 32 (100) |
| + | 10 (41) |  |  |
| ++ | 3 (12) |  |  |
| +++ | 11 (46) |  |  |
| Middle upper arm circumference (mm) Median [IQR] | 124 [114, 135] | 111 [106, 116] | 139 [135, 144] |
| Weight for height z-score Median [IQR] | -2.51 [-3.62, -1.12] | -3.63 [-4.24, -3.26] | -1.23 [-1.54, -0.82] |
| Weight for age z-score Median [IQR] | -3.57 [-4.75, -1.97] | -4.17 [-4.88, -3.74] | -1.94 [-2.22, -1.04] |
| Height for age z-score Median [IQR] | -3.43 [-4.02, -2.51] | -2.98 [-4.52, -2.26] | -1.79 [-2.17, -0.90] |

* The study triage score was adapted from the ETAT score applied in the MSF program in Niger at the time of the study. Details can be found in Supplementary table 2. Nutritional criteria (visible severe wasting, oedema on both feet and appetite test) were not included in the study triage score.

Supplementary Table 5. Urinary biochemical analysis results

|  | Kwashiorkor  (n=24) | Marasmus  (n=32) | NAM (n=32) |
| --- | --- | --- | --- |
|  |  |  |  |
| Sodium (mmol/l) |  |  |  |
| Median [min, max] | <20 [<20,245] | <20 [<20,177] | 38.1 [<20,206] |
| Potassium (mmol/l) |  |  |  |
| Median [min, max] | 4.1 [1.9,22.0] | 9.8 [1.4,53.1] | 18.7 [2.8,64.6] |
| Cl(mmol/l) |  |  |  |
| Median [min, max] | 36.6 [<20,300] | 29.6 [<20,249] | 44.9 [<20,204] |
| Albumin (mg/l) |  |  |  |
| Median [min, max] | 12.3 [1.0,105] | 28.6 [2.9,565] | 35.3 (36.4) |
| Osmolality (mOsm) |  |  |  |
| Median [min, max] | 204 [52,864] | 359 [60,874] | 474 [104,873] |
| Creatin (mg/dl) |  |  |  |
| Median [min, max] | 13.4 [2.5,71.9] | 28.6 [2.5,72.5] | 41.5 [2.5,97.8] |
| Urea (mg/dl) |  |  |  |
| Median [min, max] | 249 [43,1245] | 702 [43,2230] | 1124 [241,2815] |

Supplementary table 6. Characteristics of the subset of participants with stool samples

|  | Kwashiorkor  (n=14) | Marasmus  (n=13) | No acute malnutrition (NAM) (n=11) |
| --- | --- | --- | --- |
| Age (months) mean ± SD | 20.9± 6.1 | 20.1 ± 11.8 | 26.1 ± 12.0 |
| Sex (n female, %) | 4 (29) | 5 (38) | 2 (18) |
| Triage score* n (%) |  |  |  |
| Green (non-urgent) | 6 (42) | 4 (31) | 4 (36) |
| Yellow (priority) | 7 (50) | 8 (61) | 6 (54) |
| Red (emergency) | 1 (7) | 1 (8) | 1 (9) |
| Initial recruitment site n (%) |  |  |  |
| Children hospitalized | 8 (57) | 9 (69) | 7 (63) |
| Children in outpatient care | 6 (43) | 4 (31) | 4 (37) |
| Level of edema at baseline  n (%) |  |  |  |
| 0 | 0 (0) | 13 (100) | 11 (100) |
| + | 5 (36) |  |  |
| ++ | 3 (21) |  |  |
| +++ | 6 (43) |  |  |
| Middle upper arm circumference (mm) Median [IQR] | 127 [108, 130] | 110 [98, 116] | 140 [136, 144] |
| Weight for height z-score Median [IQR] | -2.30 [-3.76, -1.18] | -4.22 [-4.57, -3.73] | -0.99 [-1.34, -0.75] |
| Weight for age z-score Median [IQR] | -3.46 [-4.53, -2.64] | -4.25 [-5.76, -3.74] | -1.80 [-2.17, -1.10] |
| Height for age z-score Median [IQR] | -3.61 [-3.74, -  3.20] | -3.11 [-5.15, -2.01] | -1.69 [-2.19, -1.10] |

* The study triage score was adapted from the ETAT score applied in the MSF program in Niger at the time of the study. Details can be found in Supplementary table 2. Nutritional criteria (visible severe wasting, oedema on both feet and appetite test) were not included in the study triage score.

Supplementary figure 1. Sensitivity analyses for extracellular matrix protein latent variable (LV7). Removing children who tested positive for malaria rapid diagnostic test. (a) difference between kwashiorkor and non-kwashiorkor cases. (b) association with degree of oedema severity in kwashiorkor. (c) progression from baseline to treatment in kwashiorkor. Only including children with kwashiorkor who tested negative for urine protein and blood test strip. (d) difference between kwashiorkor and non-kwashiorkor cases. (e) association with degree of oedema severity in kwashiorkor. (f) progression from baseline to treatment in kwashiorkor.

Supplementary figure 2. Sensitivity analyses for extracellular matrix lipid latent variable (lLV5). Removing children who tested positive for malaria rapid diagnostic test. (a) difference between kwashiorkor and non-kwashiorkor cases. (b) association with degree of oedema severity in kwashiorkor. (c) progression from baseline to treatment in kwashiorkor. Only including children with kwashiorkor who tested negative for urine protein and blood test strip. (d) difference between kwashiorkor and non-kwashiorkor cases. (e) association with degree of oedema severity in kwashiorkor. (f) progression from baseline to treatment in kwashiorkor.

Supplementary figure 3. Component lipids of lLV5 and their levels across different nutritional phenotype and time points. Data for the individual lipids and LVs across the different groups and time points can be visualized in this link: [https://mudiboevans.shinyapps.io/MSFKwash/](https://eur03.safelinks.protection.outlook.com/?url=https%3A%2F%2Fmudiboevans.shinyapps.io%2FMSFKwash%2F&data=05%7C02%7Cbryan.gonzales%40wur.nl%7C66af4f9f088c4ec10f5908dd9c642725%7C27d137e5761f4dc1af88d26430abb18f%7C0%7C0%7C638838676157741055%7CUnknown%7CTWFpbGZsb3d8eyJFbXB0eU1hcGkiOnRydWUsIlYiOiIwLjAuMDAwMCIsIlAiOiJXaW4zMiIsIkFOIjoiTWFpbCIsIldUIjoyfQ%3D%3D%7C0%7C%7C%7C&sdata=BsaKbTdJvbB46iG3JIAnSYziNxfsAqI4Rri7kpKymN8%3D&reserved=0).

Supplementary figure 4. Heatmap showing correlation of lLV5 and its component lipids with each other; showing high positive correlation of all component lipids with lLV5.

Supplementary figure 5. Sensitivity analyses for Multi-omics integration of plasma lipidome, (semi-polar) metabolome and proteome, and clinical biochemistry results after removing children who tested positive for malaria rapid diagnostic test. a. Direct correlation among modules and clinical biochemistry parameters linked with pLV7 and lLV5. b. Direct correlation between pLV7 and lLV5. c. Plasma total protein concentration in the 3 groups (kwashiorkor, marasmus and NAM). The pink band represents children with the same total protein concentrations despite being in different groups. Partial correlation network could not be rendered due to low sample size after removing malaria positive and unknown cases.

Supplementary figure 6. Sensitivity analyses for Multi-omics integration of plasma lipidome, (semi-polar) metabolome and proteome, and clinical biochemistry results only including children with kwashiorkor who tested negative for urine protein and blood test strip. a. Partial correlation network analysis at baseline using a “spring” rendering (in *pcor* package in R). Plasma biochemical analyses: Alb – albumin; ALP – alkaline phosphatase; ALT – alanine transaminase; Blb – bilirubin; Chl – cholesterol; CRP – C-reactive protein; HDL – high density lipoprotein; Prt – total protein; TAG – total triglyceride. Point-of-care analyses (iStat): Ann – anion gap; BUN: blood urea nitrogen; Ca – ionized calcium; Cl – chloride; Crt – creatinine; Glc – glucose; Hmg – haemoglobin; Hmt – haematocrit; K – potassium; Na – sodium. b. Direct correlation among modules and clinical biochemistry parameters linked with pLV7 and lLV5. c. Direct correlation between pLV7 and lLV5. d. Plasma total protein concentration in the 3 groups (kwashiorkor, marasmus and NAM). The pink band represents children with the same total protein concentrations despite being in different groups.
